# Natural Killer Cells Dampen the Pathogenic Features of Recall Responses to Influenza Infection

**DOI:** 10.3389/fimmu.2020.00135

**Published:** 2020-02-07

**Authors:** Jason P. Mooney, Tedi Qendro, Marianne Keith, Adrian W. Philbey, Helen T. Groves, John S. Tregoning, Martin R. Goodier, Eleanor M. Riley

**Affiliations:** ^1^Department of Immunology and Infection, London School of Hygiene and Tropical Medicine, London, United Kingdom; ^2^Division of Infection and Immunity, The Roslin Institute, University of Edinburgh, Edinburgh, United Kingdom; ^3^Easter Bush Pathology, Royal (Dick) School of Veterinary Studies, University of Edinburgh, Edinburgh, United Kingdom; ^4^Department of Medicine, Imperial College London, London, United Kingdom

**Keywords:** NK cell, influenza, lung, vaccine, diphtheria toxin, mice

## Abstract

Despite evidence of augmented Natural Killer (NK) cell responses after influenza vaccination, the role of these cells in vaccine-induced immunity remains unclear. Here, we hypothesized that NK cells might increase viral clearance but possibly at the expense of increased severity of pathology. On the contrary, we found that NK cells serve a homeostatic role during influenza virus infection of vaccinated mice, allowing viral clearance with minimal pathology. Using a diphtheria toxin receptor transgenic mouse model, we were able to specifically deplete NKp46+ NK cells through the administration of diphtheria toxin. Using this model, we assessed the effect of NK cell depletion prior to influenza challenge in vaccinated and unvaccinated mice. NK-depleted, vaccinated animals lost significantly more weight after viral challenge than vaccinated NK intact animals, indicating that NK cells ameliorate disease in vaccinated animals. However, there was also a significant reduction in viral load in NK-depleted, unvaccinated animals indicating that NK cells also constrain viral clearance. Depletion of NK cells after vaccination, but 21 days before infection, did not affect viral clearance or weight loss—indicating that it is the presence of NK cells during the infection itself that promotes homeostasis. Further work is needed to identify the mechanism(s) by which NK cells regulate adaptive immunity in influenza-vaccinated animals to allow efficient and effective virus control whilst simultaneously minimizing inflammation and pathology.

## Introduction

Influenza viruses are a significant cause of respiratory tract infections, leading to seasonal epidemics and unpredictable pandemics. Globally, influenza affects ~20–30% of children and 5–10% of adults, resulting in 3–5 million cases of severe illness with up to 500,000 deaths, annually (1). The very young, old, and immunocompromised are at greatest risk of succumbing to severe illness and death (2). Currently, vaccination with live-attenuated or inactivated influenza virus is the most effective method for reducing infections at both the individual and population level (3, 4).

Although adaptive immune responses play a key role in the resolution of influenza infection, innate immune responses play an essential role in restricting virus replication and thus limiting the scale of the initial infection (5). Amongst other innate immune players, Natural Killer (NK) cells can kill virus-infected cells and help to orchestrate nascent adaptive immune responses (6). Defects in NK cells are associated with increased susceptibility to viral infections (7, 8). NK cell killing is mediated by cell surface cytotoxicity receptors (such as the natural cytotoxicity receptors NKp30, NKp44, and NKp46) binding to viral components or stress-induced ligands on the surface of virus-infected cells, leading to directed release of granzyme- and perforin-containing cytotoxic granules and/or Fas/Fas-ligand interactions (9). Additionally, cytokine (particularly interferon (IFN)-γ) production by NK cells plays an integral role in shaping adaptive immunity (10, 11). For example, NK-derived IFN-γ can activate dendritic cell migration, thus promoting T cell priming (12). Evidence for the importance of NK cells in influenza virus infection comes primarily from studies in mice, although conclusions vary depending on the precise model employed (Table S1). For example, depletion of NK cells with anti-asialo GM1 antibody in mice and hamsters resulted in increased morbidity and mortality from influenza A virus infection (13). In addition, influenza infection is lethal in mice lacking the NK cell specific receptor NKp46 (NCR1) (14), which has been reported to be a receptor for the influenza hemagglutinin (HA) protein (15, 16). Conversely, others have reported that NK cell deficiency [whether through depletion with anti-asialo GM1 or anti-NK1.1, or due to a lack of interleukin (IL)-15] reduced weight loss and increased survival (17–19). Moreover, little is known regarding the role of NK cells in vaccine-induced immunity to influenza. We set out, therefore, to address the role of NK cells during acute influenza infection, before and after vaccination, using diphtheria-toxin (DT) mediated ablation of NK cells in mice in which NKp46 expression drives co-expression of the DT receptor. Given that IL-2 produced from influenza-specific T cells is dependent on the NK cell driven IFN-γ response in early influenza infection (11, 20), we hypothesized that NK cell ablation would impair viral clearance and increase disease severity.

## Materials and Methods

### Mice

C57BL/6J and C57BL/6-Gt(ROSA)26Sor^tm1(HBEGF)Awai^/J mice were purchased from Jackson Laboratories via Charles River (Tranent, UK). C57BL/6-NKp46:iCre^+/+^ mice were kindly provided by Professor Eric Vivier (Centre d'Immunologie de Marseille-Luminy, Institut Universitaire de France, France). C57BL/6-Rosa26iDTR^+/+^ and C57BL/6-NKp46:iCre^+/+^ mice were crossed to generate F1 NKp46-iCre/Rosa26iDTR^+/−^ (referred to as NKp46-DTR). Groups of NKp46-DTR mice were age- and sex-matched for all experiments, with influenza challenge occurring between 8 and 10 weeks of age. Both sexes of the F1 generation were used in this study, with 68 female and 106 male NKp46-DTR mice being used in total. The sexes of mice used in each experiment are shown in the figure panels ({M} male, {F} female) and figure legends. All mice were maintained in individually ventilated cages in a specified pathogen free facility.

### Viral Infection and Vaccination

Egg grown influenza A/California/4/2009 virus was kindly provided by Dr. John McCauley (Crick Worldwide Influenza Centre, The Francis Crick Institute, London UK) at 128 hemagglutination units (HAU)/mL and stored at −80°C until use. For infections, mice were anesthetized by intraperitoneal (i.p.) injection of ketamine (100 mg/kg)/xylazine (10 mg/kg) and then inoculated intranasally (i.n.) with 30 μL of virus (0.5 HAU) diluted in Dulbecco's phosphate-buffered saline (DPBS) (Gibco, Loughborough UK). Mock-treated control mice were inoculated similarly with DPBS. Mice were monitored and weighed daily to assess infection and euthanized at day 4 post-infection (p.i.), or if they reached the humane endpoint of 20% loss of body weight at an earlier time point. Twenty eight days prior to challenge, some mice were vaccinated by i.p. injection of the human 2015–2016 seasonal influenza vaccine [human Sanofi-Pasteur-MSD inactivated trivalent influenza vaccine (split-virion) containing influenza haemagglutinin (HA), including A/California/7/2009 (H1N1) pdm09-like strain NYMC X-179A], diluted 1:3 in DPBS with 500 μL per mouse with a final dose of 5 μg of each HA.

### Depletion of NK Cells

For selective depletion of NK cells, NKp46-diphtheria toxin receptor (NKp46-DTR) mice were injected i.p. with 1.25 μg DT (diluted in 100 μL DPBS) on days 25 and 28 post-vaccination. Alternatively, mice were injected once with 2.5 μg DT and allowed to recover for 3 weeks prior to influenza challenge. Control (mock depleted) NKp46-DTR mice were injected i.p. with 100 μL DPBS.

### Isolation of Lung Cells and Viral Supernatant

Lungs were aseptically removed from euthanized mice and stored in 5 mL DPBS on ice until processing. Lung single-cell suspensions were obtained using methods previously described (21). Briefly, isolated lungs were cut into 2–3 mm^2^ sections and resuspended in 5 mL digestion solution of Roswell Park Memorial Institute (RPMI) 1640 Medium (Gibco) supplemented with 5 mM GlutaMax (Gibco), 5% (v/v) fetal bovine serum (Gibco), and 1% (v/v) Penicillin-Streptomycin solution (10,000 units/mL, Thermo Fisher Scientific, Loughborough UK). Digestion solutions contained DNAseI (20 μg/mL) (Roche, Basel Switzerland), and LiberaseTM (0.2 digestion units/lung) (Roche). The lung suspensions were digested at 37°C for 60 min with gentle rotating and then processed on a gentleMACS dissociator (Miltenyi, Surrey UK). Lung homogenates were then sieved through a 40 μm nylon filter to remove debris (BD Biosciences, Berkshire UK) and sieve washed with 5 mL DPBS. After centrifugation, an aliquot of lung supernatant was frozen at −70°C for downstream viral qPCR and IL-6 protein ELISA. Cell pellets were then resuspended in 5 mL ACK Lysis Buffer (Lonza, Edinburgh UK), washed and resuspended with DPBS for flow cytometry.

### Flow Cytometry

Cells were stained in 96-well U-bottom plates as described previously (22). Briefly, cell suspensions were stained with antibodies to cell surface markers, fixed (Cytofix/Cytoperm; BD Biosciences). Antibodies and dyes used for flow cytometry staining are shown in Table 1. Cells were acquired on an LSRII flow cytometer (BD Biosciences) using FACSDIVA software. Data were analyzed using FlowJo V10 (Tree Star). Gates were initially set on singlets, followed by leukocytes (removing high SSC-A and low FSC-A) and live cells. Subsequent gates were based on unstained and/or FMO controls.

**Table 1:** Antibodies and dyes used for flow cytometry.

**Antigen**	**Clone**
Zombie Aqua Dead/Live	Not applicable
CD16.2	9e9
NKp46 (CD335)	29A1.4
CD19	6D5
CD3	145.2C11
CD11b	M1/70
NK1.1	PK136
CD69	H1.2F3
CD107a	1D4B
Ly6C	HK1.4
Ly6G	1A8

### Viral Burden Determination

Influenza burden in lung-purified ribonucleic acid (RNA) was determined by one-step quantitative reverse transcription polymerase chain reaction (RT-qPCR) for the HA gene, as previously described (23). RNA was isolated from 500 μL of frozen lung cell supernatant (PureLink Viral RNA Mini Kit, Thermo). As a standard, RNA was isolated from stock virus (128 HAU/mL) and diluted 1:5 starting from 2.3 HAU equivalents per well. Purified RNA (5 μL of 25 μL elution) was mixed with 5 μL of TaqMan Fast Virus 1-step Master Mix (Thermo), 7 μL of ultra-pure water, and 1 μL each of forward primer SWH1-1080 (0.8 μM, GATGGTAGATGGATGGTACGGTTAT), reverse primer SWH1-1159 (0.8 μM, TTGTTAAGTAATYTCGTCAATGGCATT), and probe SWH1-1128 (0.25 μM, FAM-AGGATATGCAGCCGACCT-NFQMGB) with amplification conditions: 55°C, 30 min; 95°C, 2 min; 40 cycles of 95°C, 15 s and 60°C, 30 s, on a 7900HT Fast RT-PCR system (Applied Biosystems, Loughborough UK). For viral burden from whole lung tissue, 500 ng of purified RNA (as reported below) was assayed in the same manner as above.

### RNA Analysis From Tissue

Whole lung tissue was stored in RNAlater (Ambion, Loughborough UK) at post-mortem examination. RNA was extracted from tissue using RNeasy extraction kit (Qiagen, Manchester UK) according to the manufacturer's instructions, using a TissueRuptor probe (Qiagen) for tissue homogenization. RNA was then treated with DNAseI (Ambion) to remove genomic deoxyribonucleic acid (DNA) contamination. For a quantitative analysis of messenger RNA (mRNA) levels, 1 μg of total RNA from each sample was reverse transcribed in a 20 μL volume (SuperScript IV VILO Master Mix; Thermo), and 2 μL of complementary DNA (cDNA) was used for each real-time reaction. RT-qPCR was performed using the primers listed in Table 2, SYBR green (Applied Biosystems) and 7500 Real-Time PCR System (Applied Biosystems). Data were analyzed using the comparative threshold cycle (cT) method (Applied Biosystems). Target gene transcription of each sample was normalized to the respective levels of beta-Actin mRNA and represented as fold change over gene expression in control animals.

**Table 2:** Primers used for lung qPCR.

**Gene**	**Forward (Sense)**	**Reverse (Antisense)**
β-Actin	AGAGGGAAATCGTGCGTGAC	CAATAGTGATGACCTGGCCGT
IL-6	GCACAACTCTTTTCTCATTTCCACG	GCCTTCCCTACTTCACAAGTCCG
IFN-γ	CAACAGCAAGGCGAAAAAGGATGC	CCCCGAATCAGCAGCGACTCC
CXCL1	GCTTGCCTTGACCCTGAAGCTC	TGTTGTCAGAAGCCAGCGTTCAC
CXCL2	CGCCCAGACAGAAGTCATAGCCAC	TCCTTTCCAGGTCAGTTAGCCTTGC
LCN2	ACATTTGTTCCAAGCTCCAGGGC	CATGGCGAACTGGTTGTAGTCCG

### Plasma Analysis

Plasma was isolated by centrifugation of whole blood taken via cardiac puncture into a heparinized syringe and stored at −70°C. Plasma IL-6 levels were determined by sandwich enzyme-linked immunosorbent assay (ELISA) (Biolegend ELISA MAX Deluxe, London UK). Values below the blank were reported at the limit of detection for statistical purposes. To determine circulating influenza HA antibodies following influenza vaccination, a direct ELISA was done. Here, flat MaxiSorp 96-well plates (Nunc) were coated overnight with the vaccine (diluted 1:3000 in DPBS, or 0.01 μg/mL HA) or live virus (1.28 HAU/mL) at 100 μL. Using a commercial ELISA reagent kit (eBioscience catalog number BMS412, Loughborough UK), plates were washed three times and blocked for 1 h with 200 μL of assay buffer. After washing, 100 μL of serially-diluted plasma was incubated at room temperature (RT) for 2 h. After washing, 100 μL of sheep, anti-mouse immunoglobulin (Ig)-G horseradish peroxidase (HRP)-linked secondary antibody (Amersham catalog number NA931, GE Healthcare, Buckinghamshire UK) diluted 1:4000 was incubated at RT for 2 h before final washes and 3,3',5,5'-Tetramethylbenzidine (TMB) substrate development. Absorbance was determined at 450 nm by SpectraMax M5 microplate reader (Molecular Devices, Wokingham UK). Vaccine take was confirmed for all vaccinated mice with HA-IgG ELISA at 1:100 dilution of plasma.

### Histopathology

Sections were cut at 4 μm thickness from formalin-fixed, paraffin-embedded whole lung tissues and stained with hematoxylin and eosin (H&E) by the University of Edinburgh Easter Bush Pathology Laboratory and scored, in a blinded fashion, for inflammation (vasculitis, bronchiolitis, and alveolitis), oedema (perivascular oedema, peribronchiolar oedema, and alveolar oedema), leukocyte infiltration (perivascular, peribronchiolar, and in alveolar walls) and neutrophil infiltration (perivascular, peribronchiolar, and in alveolar walls). Each criterion was scored numerically as unremarkable (0), mild (1), mild to moderate (2), moderate (3), moderate to marked (4), and marked (5). Photomicrographs of histological sections were taken using a 20× objective with an Olympus BX41 microscope using an Olympus DP72 camera.

### Statistical Analysis

Differences between groups were analyzed by Mann-Whitney U test on compiled data from at least two independent experiments, unless otherwise noted in legends. Further, data from each independent experiment is shown as supplementary data in Figures S1–S10. Spearman's correlation was used to identify statistically significant associations between weight loss, influenza burden and lung neutrophils. *P* values above 0.1 are displayed as “ns” (not significant) in the figures, with values below 0.05 considered significant. All analyses were conducted using GraphPad Prism 7.

## Results

### Influenza Vaccination Reduces Weight Loss and Viral Burden in Mice

**Figure 1:**
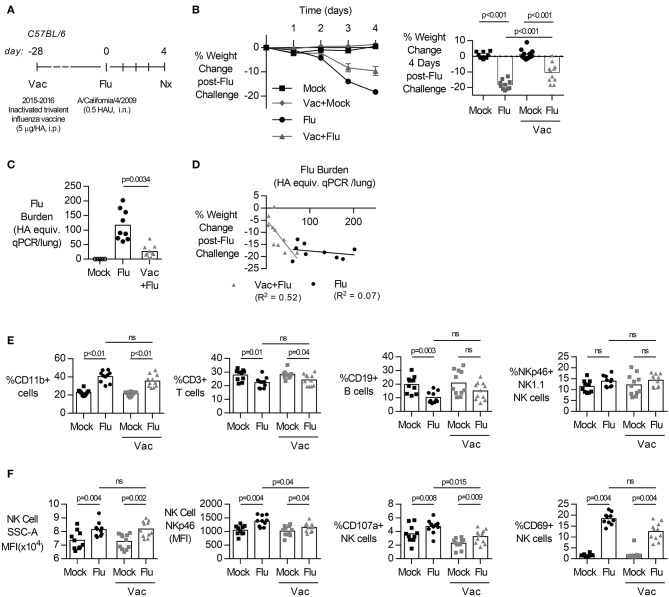
Primary influenza infection induces rapid weight loss and NK cell activation in lung but vaccination reduces weight loss and lung viral burden. **(A)** C57BL/6 female mice were challenged intranasally with 0.5 hemagglutination units (HAU) of influenza A/California/4/2009 (Flu) or mock treated with DPBS (Mock). Four weeks prior to challenge, mice were vaccinated intraperitoneally with the trivalent Sanofi influenza vaccine (Vac). **(B)** Weight loss (mean ± SEM) over 4 days. **(B–F)** At day 4 post infection, lungs were excised and cell-free supernatant was analyzed by qPCR for influenza viral burden (plotted against a dose curve of Flu with known HAU, giving HAU equivalents) **(C)** and plotted against weight loss **(D)**. Data fitted to a non-linear regression line with R square value shown **(D)**. **(E,F)** Lung cell pellets were analyzed by flow cytometry for **(E)** cellular abundance and **(F)** Natural Killer (NK) activation markers. Data is compiled from two independent experiment (*n* = 5–11/group), with each independent experimental data shown in Figures S1, S2. Dots represent individual mice with bars showing mean. Significance determined by Mann–Whitney *U*-test, ns, not significant.

To characterize the role of NK cells in influenza infection and immunization, we established a model of acute influenza infection in C57BL/6J mice (Figure 1). C57BL/6J mice were infected i.n. with 0.5 HAU of influenza strain A/California/04/2009. Infected mice developed an acute infection, losing 20% of their body weight by 4 days post-infection (Figure 1A). Mice were also vaccinated, intraperitoneally with the human Sanofi-Pasteur-MSD inactivated trivalent influenza vaccine (split-virion), 4 weeks prior to influenza challenge. Vaccinated mice lost significantly less weight (Figure 1B) and had lower viral burden in their lungs (Figure 1C) compared to unvaccinated mice; the reduction in influenza burden in the lung correlated with reduced weight loss (Figure 1D).

Acute influenza infection was marked by a significant increase in the frequency of CD11b+ cells in the lung, with a decrease in proportions of T (%CD3+) (Figure 1E, gating Figure S12). The frequency of NK cells, identified by NK1.1 and NKp46 co-expression (19, 24), did not change after influenza infection (Figure 1E). However, there was a significant increase in the side-scatter (SSC) of the NK cell population (Figure 1F, gating Figure S13), indicating increased activation (25–27) and expression of NKp46, an activating NK cell receptor reported to bind influenza-derived HA (14, 28, 29), was also increased in infected animals. Lastly, there was a significant increase in the frequency of NK cell activation marker CD69 (Figure 1F). Together, these results suggest that the immune response to acute influenza infection, whether in a naïve or vaccinated animal, is characterized by an influx of CD11b+ cells and activated NK cells.

### Depletion of NK Cells Prior to Influenza Challenge Reduces Lung Virus Burden but Increases Weight Loss in Vaccinated Mice

**Figure 2:**
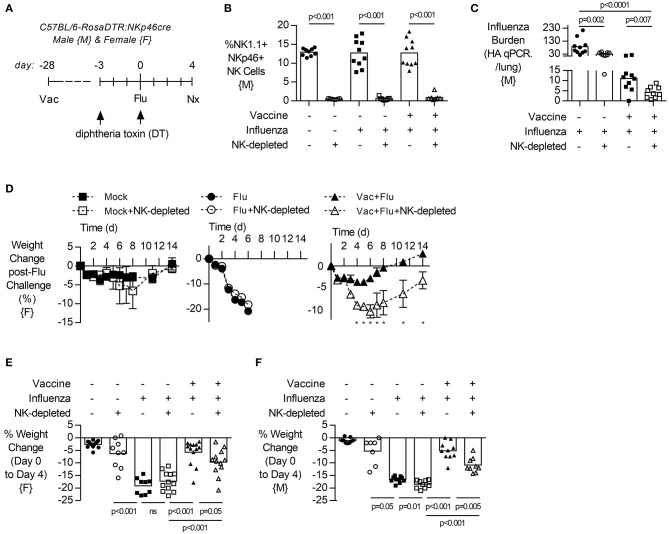
NK cell depletion reduces lung viral burden and increases weight loss in vaccinated, influenza-challenged mice. Transgenic C57BL/6 mice with NKp46 driven expression of diphtheria toxin (DT) receptor were vaccinated 28 days prior to intranasal influenza (flu) challenge, as in Figure 1. **(A)** Immediately prior to infection, a subset of mice received two intraperitoneal injections of DT (1.25 μg). **(B)** Levels of NK1.1+, NKp46+ NK cells in the lung, as a proportion of singlet, live leukocytes at 4 days post infection (necropsy, nx). **(C)** At 4 days post infection, lung cell-free supernatants were analyzed by qPCR for influenza viral burden (plotted against a dose curve of IFA with known HAU, giving HAU equivalents). **(D)** Weight loss (mean ± SEM) followed for 14 days post challenge, one experiment (*n* = 4–5/group) **(E–F)** Weight loss at day 4 post challenge. Data from males {M} (*n* = 9–10/group) is compiled from two independent experiments and females {F} (*n* = 9–13/group) from three independent experiments, with each independent experimental data shown in Figure S3. Dots represent individual mice with bars showing mean. Significance determined by Mann–Whitney *U*-test, ns, not significant. ^*^p < 0.05.

To evaluate the contribution of NK cells to vaccine-induced protection from influenza infection, both male and female transgenic mice (NKp46-DTR) were selectively depleted of NK cells by injection of DT (Figure 2A) 3 days before infection. DT treatment resulted in a robust depletion of NK cells from the lung (Figure 2B, males, gating Figure S14, and females Figure S9) but did not change circulating antibodies established by vaccination (Figure S11, both sexes).

Depletion of NK cells prior to influenza challenge infection led to a significant decrease in influenza burden in the lung in vaccinated animals (Figure 2C, males). In pilot experiments, the humane endpoint of 20% weight loss in unvaccinated mice was reached by 6 days post infection (Figure 2D, females). However, vaccinated animals lost only 5% of their body weight and recovered to pre-infection weights by day 8 (Figure 2D). Interestingly, vaccinated and infected animals which lacked NK cells had prolonged weight loss which was more severe (10%) than in NK cell-intact vaccinated mice (5%) and recovered to baseline only by day 14 (Figure 2D). This exacerbated weight loss of infected NK-depleted animals at 4 days post infection compared to NK-cell sufficient mice was seen in both sexes (Figure 2E, female, and Figure 2F, male).

### No Change in Lung Inflammatory Cytokine Response to Influenza Upon NK Cell Depletion

**Figure 3:**
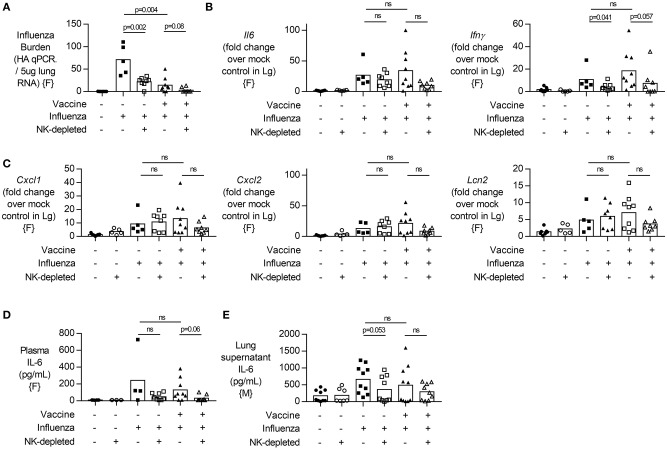
NK cell depletion in vaccinated, influenza-challenged mice does not change inflammatory transcripts. Four days post infection in the model described in Figure 2A, **(A)** Lung RNA was analyzed by qPCR for influenza viral burden (plotted against a dose curve of influenza with known HAU, giving HAU equivalents per 5 μg RNA tested). **(B,C)** Transcript levels of inflammatory cytokine genes **(B)** *Il6* and *Ifn*γ and **(C)** neutrophil-related chemokines *Cxcl1* and *Cxcl2*, along with neutrophil lipocalin protein (*Lcn2*). RNA induction normalized to housekeeping gene β-actin and displayed as induction over mock-treated control mice. **(D)** Plasma levels of IL-6 (pg/mL). **(E)** IL-6 levels in lung supernatants after whole lung enzymatic digestion for single cell isolation and viral burden quantification. Data from females {F} (*n* = 5–9/group) and males {M} (*n* = 7–10/group) is compiled from two independent experiments, with each independent experimental data shown in Figures S4, S5. Dots represent individual mice with bars showing mean. Significance determined by Mann–Whitney *U*-test, ns, not significant.

Given that vaccinated NK cell-depleted animals lost more weight than vaccinated NK cell-intact animals despite a lower viral burden (Figure 2), we next looked at inflammatory markers in the lung and plasma. In agreement with data from lung supernatants (Figure 2C, males), viral burden determined from whole lung RNA preparations was significantly lower after NK cell depletion in unvaccinated animals, and not in vaccinated animals (*p* = 0.08) (Figure 3A, females). While *Il6* and *Ifn*γ expression were increased with influenza infection (Figure 3B), regardless of vaccination status and viral burden (Figure 3A), transcripts of *Il6* were not significantly changed in NK cell depleted mice, while *Ifn*γ showed a trend for a decrease in NK cell depleted mice (Figure 3B).

Given that neutrophil influx into an influenza-infected lung can enhance viral control (30), but can also contribute to tissue damage through the release of extracellular traps (31), we wondered whether increased neutrophil activity in NK cell-depleted mice might explain our findings. We therefore determined transcript levels of neutrophil chemokines (*Cxcl1, Cxcl2)* and neutrophil secreted protein *Lcn2* but found no significant differences in transcript concentrations between NK cell-deficient and NK cell-intact animals (Figure 3C). Lastly, we assessed circulating levels of IL-6 [which have been implicated in neutrophil-mediated effects (30)] and found no significant difference in plasma or lung supernatant IL-6 concentration in either vaccinated or unvaccinated NK-depleted mice compared to NK cell-intact mice (Figures 3D,E).

### Influenza-Mediated Lung Pathology in Vaccinated and NK Cell-Depleted Mice

**Figure 4:**
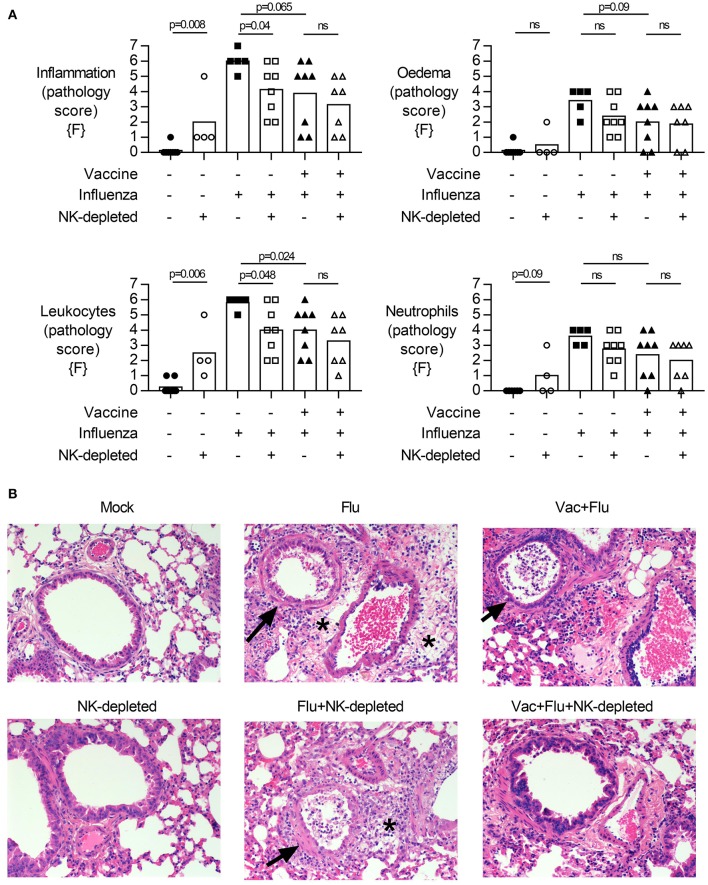
NK cell depletion in vaccinated, influenza-challenged mice does not alter pathology. Four days post infection in the model described in Figure 2A, whole lungs were excised, stored in 10% formalin, and embedded on paraffin for hematoxylin and eosin staining. Pathology was scored for: **(A)** Inflammation (vasculitis, bronchiolitis, and alveolitis), Edema (perivascular, peribronchiolar, and alveolar), Leukocytes and Neutrophils (in perivascular space, peribronchiolar space, and alveolar wall). Full scoring details in Supplementary Files. Pathology scores from females {F} (*n* = 4–8/group) is compiled from two independent experiments, with each independent experimental data shown in Figure S6. Dots represent individual female {F} mice with bars showing mean. Significance determined by Mann–Whitney test, ns, not significant. **(B)** Representative photomicrographs of pathological changes observed. Arrows, bronchiolitis with exudates in bronchiolar lumina. ^*^Perivascular and peribronchiolar edema. Scale bar in mock equals 50 μm.

Given the decreased viral burden but increased weight loss upon influenza challenge in vaccinated NK cell-depleted mice compared to NK cell-sufficient mice (Figure 2E, males and 3A, females), we examined the lungs for histological evidence of pathology. H&E stained lung sections were assessed for pulmonary inflammation (vasculitis, bronchiolitis, and alveolitis), edema (perivascular, peribronchiolar, and alveolar), and infiltrating lymphocytes and neutrophils (perivascular, peribronchiolar, and alveolar) (Figure 4, females; see Datasheet 2 for individual mouse data). Influenza infection alone induces severe pathology (Figure 4). Interestingly, while weight loss was more severe in vaccinated and influenza challenged NK-depleted mice than in NK cell-sufficient mice, pathological changes in the lungs were significantly different only between unvaccinated and NK-depleted mice—and not between vaccinated and NK-depleted mice (Figure 4).

**Figure 5:**
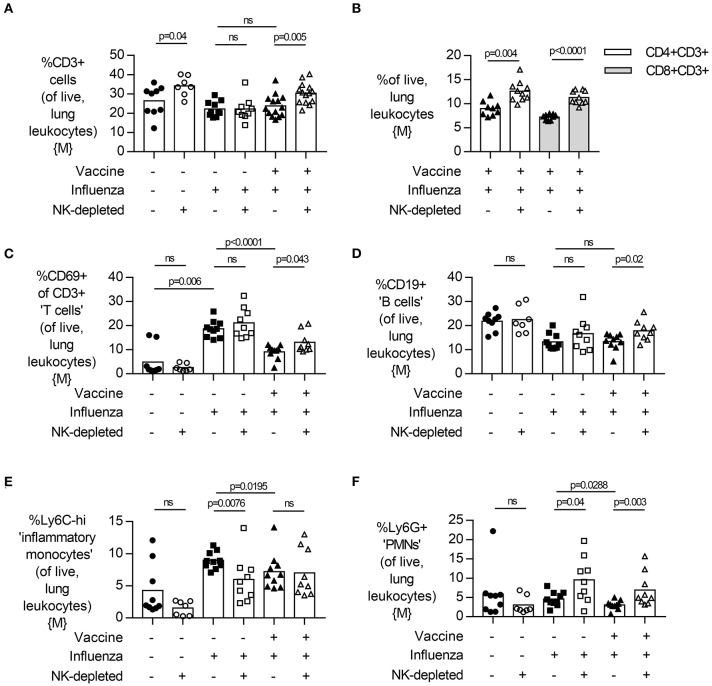
NK cell depletion in vaccinated, influenza-challenged mice increases lung CD3+ T cell and neutrophil infiltration. Four days post infection in the model described in Figure 2A, whole lungs were excised and single cells isolated for flow cytometry. Proportion (%) of **(A)** CD3+ T cells, **(B)** CD3+CD4+ and CD3+CD8+ T cells, and **(C)** active (CD69+) CD3+ T cells, as determined from singlet, live lung leukocytes. Proportion (%) of **(B)** CD19+ B cells, **(C)** Ly6C-high inflammatory monocytes, and **(D)** Ly6G+ neutrophils. Data in **(A)** from males {M} (*n* = 7–14/group) is compiled from three independent experiments and data in **(B–F)** from males {M} (*n* = 7–10/group) is compiled from two independent experiments, with each independent experimental data shown in Figure S9. Dots represent individual mice, with bar representing mean. Significance determined by Mann-Whitney *U* test, ns, not significant.

Finally, we measured the relative frequency of infiltrating leukocytes and neutrophils in the lung by flow cytometry (Figure 5, males). Influenza infection led to an infiltration of CD3+ T cells (Figure 5A, gating Figure S15) into the lung of NK-depleted mice, both CD4+ and CD8+ (Figure 5B). Infection of unvaccinated mice was associated with an increase in the frequency of activated (CD69+) T cells in the lung (*p* = 0.006) (Figure 5C); this was attenuated by vaccination (*p* < 0.001) and increased by depletion of NK cells. There were no obvious effects of infection or vaccination on the frequency of CD19+ B cell populations in the lung; the modest apparent increase in B cell proportions in NK cell-depleted mice (*p* = 0.02) may simply reflect distortion of cell percentages by the absence of NK cells (Figure 5D, gating Figure S16). However, given that influenza infection increases the influx of CD11b+ cells into the lung (Figure 1D), we further characterized influx of both inflammatory monocytes (%Ly6C-high) and neutrophils (%Ly6G+) in the lungs of NK-depleted mice. The modest reduction in leukocyte infiltration in the lung that was evident from histology (Figure 4), was reflected in reduced infiltration of inflammatory monocytes into the lung in unvaccinated NK cell-sufficient animals compared to NK-depleted animals (Figure 5E), but there was no additional effect of combining vaccination and NK cell depletion. Neutrophil infiltration into the lung was also increased with NK cell depletion, with the frequency of Ly6G+ polymorphonuclear cells being significantly higher in both vaccinated and unvaccinated influenza-infected NK cell-depleted mice compared to NK cell sufficient mice (Figure 5F). A single experiment in female mice also found a trend for an increase in Ly6G+ neutrophils in vaccinated and NK cell-depleted mice (Figure S9). Taken together, these data suggest that the increased weight loss observed with influenza infection in NK cell-depleted vaccinated mice compared to NK cell intact vaccinated mice may be due, in part, to failure of NK cell-depleted mice to control cellular infiltration into the lungs, despite reduced viral burden.

### Depletion of NK Cells After Vaccination, and Subsequent Repopulation, Does Not Alter the Response to Influenza Challenge in Mice

**Figure 6:**
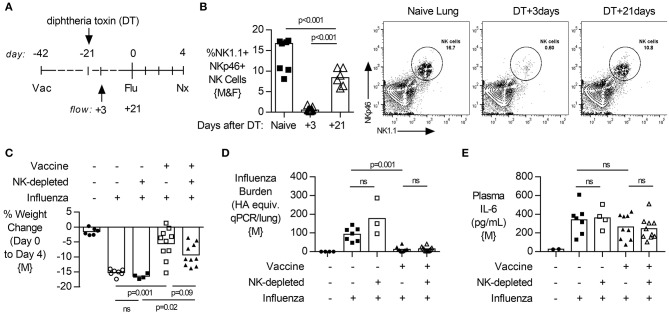
Depletion of NK cells after vaccination, and subsequent repopulation, does not alter lung viral burden, disease severity or systemic inflammation after challenge. **(A)** Transgenic C57BL/6 mice with NKp46 driven expression of diphtheria toxin (DT) receptor were vaccinated 42 days (d) prior to intranasal influenza (Flu) challenge and treated with DT (NK-depleted) 21 days prior to challenge with necropsy (nx) at 4 days post influenza challenge. **(B)** At 3 and 21 days post DT treatment, lungs were excised and single cells isolated for flow cytometry for the proportion (%) of NK1.1+, NKp46+ NK cells. **(C)** Weight loss at 4 days post influenza challenge. **(D)** Lung cell-free supernatants were analyzed by qPCR for influenza viral burden (plotted against a dose curve of Flu with known HAU, giving HAU equivalents). **(E)** Plasma levels of IL-6 (pg/mL). Data from males {M} (*n* = 4–10/group) is compiled from two independent experiments, with each independent experimental data shown in Figure S10. Dots represent individual mice with bars showing mean. Significance determined by Mann-Whitney test, ns, not significant.

To determine if the effects of NK cell depletion on post vaccination immunity to influenza were mediated by NK cells present at (and potentially affected by) vaccination (32), we depleted NK cells (by a single treatment with 2.5 μg of DT) 3 weeks after influenza vaccination (as previously) but then waited another 3 weeks before challenging the mice (to allow repopulation) (Figure 6A). NK cells were initially (after 3 days) very effectively depleted by the DT treatment and, as predicted, the NK cell compartment did partially recover by the time of influenza challenge (Figure 6B). In this case, removal of NK cells present at vaccination and subsequent NK cell repopulation resulted in influenza infections that were not significantly different from those in intact mice, with no significant differences in disease severity (weight loss; Figure 6C), lung viral load (Figure 6D) or circulating IL-6 concentrations (Figure 6E). This experiment suggests that the effects of NK cell depletion on post vaccination immunity are due to the lack of all NK cells, rather than a lack of NK cell populations that were primed or activated by vaccination.

## Discussion

**Figure 7:**
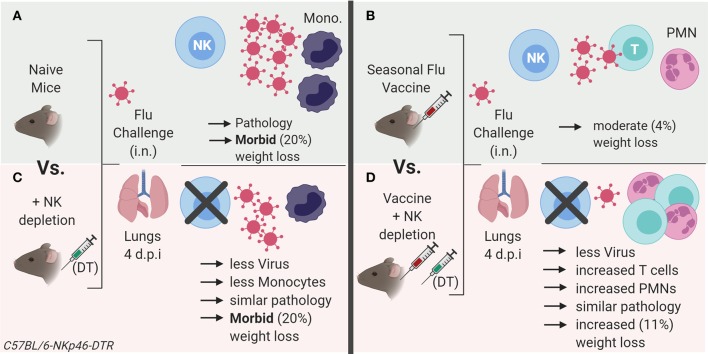
Summary model of role of NK cells in influenza vaccination and challenge. **(A)** Influenza virus infection in naïve mice results in an influx of inflammatory monocytes (Ly6C^high^) into the lung, with evident histopathology and rapid weight loss. **(B)** Vaccination reduces lung virus load and associated pathology (weight loss). **(C)** In unvaccinated animals, NK cell depletion reduces viral load and associated inflammation after influenza infection compared to NK cell replete animals, but animals still lose a significant amount of weight. **(D)** NK depletion of vaccinated mice further reduces influenza viral load and weight loss after infection, yet pathology scores are similar to those of NK intact mice. However, vaccination combined with NK depletion is accompanied by an increase in infiltration of neutrophils and activated T cells into the lung after influenza infection, suggesting that NK cell depletion enhances the control of virus replication as a result of enhanced adaptive immunity but that this comes at the price of significantly increased morbidity. Model created with BioRender.

The key findings of this study, summarized in Figure 7, are that: (1) influenza vaccination is effective in reducing viral burden and weight loss in mice; (2) in both vaccinated and unvaccinated mice, NK cells ameliorate disease (weight loss) at the expense of delaying viral clearance; (3) that the magnitude of the effect on disease is greater in vaccinated than unvaccinated mice (2.5-fold greater weight loss vs. 1.11-fold, respectively), and (4) the depletion of any “memory” NK cells that might have been induced by vaccination did not alter the response to influenza virus in vaccinated mice. These data suggest that NK cells play an important homeostatic role, allowing influenza virus to be controlled without causing severe disease, and that this effect is enhanced by vaccination—indicating a role for NK cells in regulating the adaptive immune response. Our study therefore supports previous data suggesting an important role for NK cells in moderating adaptive immune effector mechanisms (33).

Previous work has suggested that the role of NK cells during influenza infection is dependent on the infecting dose of the virus, with NK cells being protective during low dose infection (0.5 HAU) and pathogenic during high dose infection (5 HAU) (19). Although we used the A/California/4/2009 strain of influenza rather than PR8 strain used by Zhou et al. we used an infecting dose that is similar to the low dose used by Zhou et al. (0.5 HAU) and saw a similar trend. Interestingly, an opposite effect was seen during murine cytomegalovirus (MCMV) infection where NK cells prevented pathology at high viral doses but enhanced disease at low doses (34).

Although we saw a clear impact of NK cell depletion on the post-vaccination response to influenza in both male and female mice, we did observe some sex-specific differences in our data. Whilst we do not have a complete, replicated set of all data in both sexes at every time point, limiting our ability to draw specific conclusions regarding the impact of sex on the role of NK cells during influenza infection and vaccination, we found that NK cell-depleted unvaccinated male mice (but not female mice) lost significantly more weight than intact mice after infection (Figures 2E,F) despite the two sexes having similar viral loads. We also observed that female mice generated higher titres of neutralizing antibodies after influenza challenge (Figure S11C), confirming previous work with PR8 virus by Lorenzo et al. (35). Sex differences in response to influenza infection and influenza vaccination have been noted in mice and in humans [reviewed by (36)] however, many previous studies to determine the role of NK cells during acute influenza infection mice have used male or female mice only, or have failed to define the sex of the mice used (Table S1). Future work should consider the impact of sex on the role of NK cells during a recall response to viral infection.

The mechanisms by which NK cells moderate adaptive immune responses are not fully understood. Our observation that NK cell depletion tended to increase neutrophil influx into the lungs of influenza infected mice, and significantly increased neutrophil accumulation in lungs of vaccinated mice, suggests that NK cells may serve to limit neutrophil migration to sites of infection. This would be in line with studies showing that neutrophils are important in controlling influenza virus infection (37–39) but can contribute to severe pulmonary pathology (40–42).

Another possibility is that NK cells regulate the accumulation of adaptive, cytotoxic (CD8+) effector T cells at the site of infection, thereby reducing tissue damage but slowing viral clearance. In RSV infection, for example, CD8+ cells are directly associated with weight loss and depleting them reduces disease (43). NK cells have been shown to eliminate activated CD4+ and CD8+ T cells in different model systems (33, 34, 44), mediated by either perforin or TNF-related apoptosis-inducing ligand (TRAIL) (45, 46). However, thus far, in influenza vaccination models CD8+ T cells appear to contribute to both reduced viral load and reduced disease severity (47). Due to limitations of cell numbers, we were unable to characterize the responsiveness of T cells in this study and therefore further work is needed to determine whether the lack of NK cells removes the brake on adaptive T cell responses during influenza infection.

In a recent study (48), murine NK cells licensed on self MHC were shown to localize to infected lung tissue and produce IFN-γ after influenza A (strain PR8) infection. Unlicensed NK cells, in contrast, were enriched in draining (mediastinal) lymph nodes, produced GM-CSF and promoted dendritic cell infiltration and CD8+ T cell responses. It is therefore likely that distinct subsets of NK cells may selectively promote inflammation or antiviral immunity. Furthermore, this may differ depending on levels of adaptive immunity.

In humans, memory-like NK cells can be generated by cytokine or influenza virus pre-activation; these cells show enhanced responses to cytokines or influenza virus upon re-stimulation (11, 32, 49). It is conceivable that prior activation of NK cells, for example by vaccine-induced inflammatory cytokines, could influence the migration and function of these cells upon viral challenge. Certainly, human nasal challenge with Fluenz induces a local (nasal mucosal) innate cytokine signature with potential NK cell activating capacity (50). Nevertheless, we wondered whether a similar phenomenon might also be apparent in this mouse model (where more precise dissection of the underlying local mechanism of NK cell enhancement might be possible). Therefore, we depleted NK cells immediately after vaccination (thus removing any putative “memory” NK cells, defined as cells with altered function after prior exposure), and rested the mice for 3 weeks to allow repopulation before challenging with influenza virus. We observed no differences in the outcome of influenza infection between these NK cell-depleted/repopulated vaccinated mice and intact, vaccinated mice. These data suggest that “memory” NK cells are not induced by influenza vaccination in mice or that any such cells are no more effective at moderating adaptive responses to influenza than are “naïve” NK cells.

In mice, cytokine-induced memory-like NK cells can be adoptively transferred and maintained by homeostatic proliferation (51) and work by Li et al. has demonstrated that NK cells generated in the liver following primary infection can reduce influenza viral burden in the lung upon secondary challenge in *Rag1*-deficient mice (52). However, to date, memory-like NK cells have not been demonstrated after influenza vaccination and the contribution of either tissue-resident or blood-derived “memory” NK cells during a robust recall response in B/T-cell intact mice has yet to be characterized. In addition, human studies have been limited to peripheral blood and further investigations are needed to test how NK cells influence inflammatory cellular infiltrates at the site of virus infection. Vaccination and challenge studies (with local mucosal sampling for virus, cellular infiltrates and cytokine production) are now needed to reveal the impact of vaccine-induced NK cell priming and “memory” generation on virus replication and pathology.

There are some important caveats to keep in mind, however. Firstly, in contrast to previous studies using mice lacking NK cells at the time of infection or vaccination, or depleted of NK cells with antibodies on animals of different sexes (Table S1), we used an inducible, endogenous method of NK cell depletion which may have fewer limitations (24). Nevertheless, DT treatment alone lead to some limited weight loss and pulmonary pathology, likely due to death of ~15% of lung leukocytes, although there was no induction of inflammatory cytokines nor any influx of immune cells into the lung. It remains a possibility, therefore, that the mild inflammation induced by NK cell depletion may have affected the outcome of the experiments. Further, sex differences in influenza-mediated lung pathology have been noted (36). Secondly, the ethically-approved humane end point for our study was deemed to be 20% weight loss (after which animals were euthanized), essentially precluding determination of long-term survival after NK cell depletion but some studies have shown only limited correlation between weight loss and long term survival (18), with viral challenge dose a greater predictor of survival despite similar weight loss kinetics (19). A further caveat of the Rosa-DT conditional “NK cell” depletion system used here is a potential parallel depletion of mature NKp46+ ILC1 and ILC3 cells (53). Studies in deficient *ncr1*^gfp^ mice, which are susceptible to lethal influenza A infection, demonstrate systemic loss of ILC1, whilst maintaining NKp46 negative NK cell subsets (14, 54). Although rare in the non-pathologic lung, our studies do therefore not exclude a potential contribution of ILC-1s to inflammatory processes after influenza vaccination or challenge infection. Lastly, characterization of cytokine protein levels in the bronchoalveolar lavage fluid would be beneficial in understanding transcriptional changes observed in this study.

To summarize, we have demonstrated that NK cells play a homeostatic role in adaptive immunity to influenza infection in mice. While the precise mechanism by which NK cells modulate adaptive immunity remains unclear, their presence is crucial for resolving infection with minimal immune pathology. Further studies to determine the mechanism(s) at play may inform the design of safer and more effective influenza vaccines.

## Data Availability Statement

The raw data supporting the conclusions of this article will be made available by the authors, without undue reservation, to any qualified researcher.

## Ethics Statement

The animal study was reviewed and approved by the animal welfare and ethical review board of the London School of Hygiene and Tropical Medicine (LSHTM). All experiments were performed in accordance with United Kingdom (UK) Home Office Regulations under Project License 70/8291.

## Author Contributions

Study concept and design: JM, MG, and ER. Data generation and analysis: JM, TQ, MK, AP, and HG. Drafting and revision of manuscript: JM, TQ, MK, AP, JT, MG, and ER. Critical appraisal and approval for submission: all authors.

### Conflict of Interest

The authors declare that the research was conducted in the absence of any commercial or financial relationships that could be construed as a potential conflict of interest.
