# Supplementary material for: Natural Killer Cells Dampen the Pathogenic Features of Recall Responses to Influenza Infection
Source: Front Immunol. 2020 Feb 7;11:135. doi: 10.3389/fimmu.2020.00135 (PMC7019041; doi:10.3389/fimmu.2020.00135)

**Supplementary Digital Content:**

**Natural Killer cells dampen the pathogenic features of recall responses to influenza infection.**

Jason P. Mooney^1,2^; Tedi Qendro^1^; Marianne Keith^2^; Adrian W. Philbey^3^; Helen T. Groves^4^; John S. Tregoning^4^; Martin R. Goodier^1^; Eleanor M. Riley^1,2^

Supplementary Figure Legends:

**Figure S1, related to Figure 1.** C57BL/6 female mice were challenged intranasally with 5 hemagglutination units (HAU) of influenza A/California/4/2009 (Flu) or mock treated with DPBS (Mock). Four weeks prior to challenge, mice were vaccinated intraperitoneally with the trivalent Sanofi influenza vaccine (Vac), with 5ug of HA. **(A-B)** Weight loss over 4 days post influenza challenge. **(C)** At day 4 post infection, lungs were excised and cell-free supernatant was analyzed by qPCR for influenza viral burden (plotted against a dose curve of Flu with known HAU, giving HAU equivalents) and plotted against weight loss **(D)**. Data fitted to a non-linear regression line with R square value shown **(D)**. **(E)** Similar to S1A, mice were vaccinated with either 7.5ug or 12ug of HA, challenged with 5 HAU of Flu and followed over 4 days for weight loss. Data is from representative experiments. Dots represent individual mice with bars showing mean. Line data in A shown as mean±SEM. Significance determined by Mann-Whitney U test, ns = not significant.

**Figure S2, related to Figure 1.** C57BL/6 female mice were challenged intranasally with 5 hemagglutination units (HAU) of influenza A/California/4/2009 (Flu) or mock treated with DPBS (Mock). Four weeks prior to challenge, mice were vaccinated intraperitoneally with the trivalent Sanofi influenza vaccine (Vac). At day 4 post infection, lungs were excised and single-cell pellets were analyzed by flow cytometry for **(A)** cellular abundance and **(B)** Natural Killer (NK) activation markers. Data is from representative experiments. Dots represent individual mice with bars showing mean. Line data in A shown as mean±SEM. Significance determined by Mann-Whitney U test, ns = not significant.

**Figure S3, related to Figure 2.** Transgenic C57BL/6 mice with NKp46 driven expression of diphtheria toxin (DT) receptor were vaccinated 28 days prior to intranasal influenza (flu) challenge, as in Fig. 1. Immediately prior to infection, a subset of mice received two intraperitoneal injections of DT (1.25 μg). **(A)** Levels of NK1.1+, NKp46+ NK cells in the lung, as a proportion of singlet, live leukocytes at 4 days post infection. **(B)** At 4 days post infection, lung cell-free supernatants were analyzed by qPCR for influenza viral burden (plotted against a dose curve of IFA with known HAU, giving HAU equivalents). **(C-D)** Weight loss at day 4 in male (C) or female (D) mice. Data is from representative experiments. Dots represent individual mice with bars showing mean. Significance determined by Mann-Whitney U test, ns = not significant.

**Figure S4, related to Figure 3.** Four days post infection in the model described in Fig. 2A, **(A)** Lung RNA was analyzed by qPCR for influenza viral burden (plotted against a dose curve of influenza with known HAU, giving HAU equivalents per 5ug RNA tested). **(B-C)** Transcript levels of inflammatory cytokine genes **(B)** *Il6* and **(C)** *Ifnγ*. RNA induction normalized to housekeeping gene β-actin and displayed as induction over mock-treated control mice. **(D)** Plasma levels of IL-6 (pg/mL). Data is from representative experiments. Dots represent individual female {F} mice with bars showing mean. Significance determined by Mann-Whitney U test, ns = not significant.

**Figure S5, related to Figure 3.** Four days post infection in the model described in Fig. 2A, **(A-C)** Transcript levels of neutrophil-related chemokines **(A)** *Cxcl1* and **(B)** *Cxcl2*, along with neutrophil lipocalin protein **(C)** (*Lcn2*). RNA induction normalized to housekeeping gene β-actin and displayed as induction over mock-treated control mice. Data is from representative experiments. Dots represent individual female {F} mice with bars showing mean. Significance determined by Mann-Whitney U test, ns = not significant.

**Figure S6, related to Figure 4.** Four days post infection in the model described in Fig. 2A, whole lungs were excised, stored in 10% formalin, and embedded on paraffin for hematoxylin and eosin staining. Pathology was scored for: **(A)** Inflammation (vasculitis, bronchiolitis, and alveolitis), **(B)** Oedema (perivascular, peribronchiolar, and alveolar), **(C)** Leukocytes and **(D)** Neutrophils (in perivascular space, peribronchiolar space, and alveolar wall). Full scoring details in supplementary files. Data is from representative experiments. Dots represent individual female {F} mice with bars showing mean. Significance determined by Mann-Whitney test, ns = not significant.

**Figure S7, related to Figure 5.** Four days post infection in the model described in Fig. 2A, whole lungs were excised and single cells isolated for flow cytometry. **(A)** Proportion (%) of CD3+ T cells. Data is from representative experiments. Dots represent individual male {M} mice with bars showing mean. Significance determined by Mann-Whitney test, ns = not significant.

**Figure S8, related to Figure 5.** Four days post infection in the model described in Fig. 2A, whole lungs were excised and single cells isolated for flow cytometry. Proportion (%) of **(A)** CD3+CD4+ and CD3+CD8+ T cells, and **(B)** active (CD69+) CD3+ T cells, as determined from singlet, live lung leukocytes. Proportion (%) of **(C)** CD19+ B cells, **(D)** Ly6C-high inflammatory monocytes, and **(E)** Ly6G+ neutrophils. Data is from representative experiments. Dots represent individual male {M} mice with bars showing mean. Significance determined by Mann-Whitney test, ns = not significant.

**Figure S9, related to Figure 5.** Transgenic female {F} C57BL/6 mice with NKp46 driven expression of diphtheria toxin (DT) receptor were vaccinated 28 days prior to intranasal influenza (flu) challenge, as in Fig. 2A. Immediately prior to infection, a subset of mice received two intraperitoneal injections of DT (1.25 μg). **(A)** Levels of NK1.1+, NKp46+ NK cells in the lung, as a proportion of singlet, live leukocytes at 4 days post infection. **(B)** At 4 days post infection, lung cell-free supernatants were analyzed by qPCR for influenza viral burden (plotted against a dose curve of IFA with known HAU, giving HAU equivalents). Four days post infection, whole lungs were excised and single cells isolated for flow cytometry. Proportion (%) of **(C)** CD19+ B cells, **(D)** Ly6C-high inflammatory monocytes, and **(E)** Ly6G+ neutrophils. Data is from one representative experiment (left panels, n=5/group). Dots represent individual female {F} mice with bars showing mean. Significance determined by Mann-Whitney test, ns = not significant.

**Figure S10, related to Figure 6.** Transgenic C57BL/6 mice with NKp46 driven expression of diphtheria toxin (DT) receptor were vaccinated 42 days (d) prior to intranasal influenza (Flu) challenge and treated with DT (NK-depleted) 21 days prior to challenge with necropsy (nx) at 4 days post influenza challenge. **(A)** At 3 and 21 days post DT treatment, lungs were excised and single cells isolated for flow cytometry for the proportion (%) of NK1.1+, NKp46+ NK cells in female {F} mice. **(B-D)** At day 21 post DT treatment, male {M} mice were challenged with influenza and **(B)** weight loss was measured at 4 days post challenge. **(C)** Lung cell-free supernatants were analyzed by qPCR for influenza viral burden (plotted against a dose curve of Flu with known HAU, giving HAU equivalents). **(F)** Plasma levels of IL-6 (pg/mL). Data is from representative experiments. Dots represent individual male mice with bars showing mean. Significance determined by Mann-Whitney test, ns = not significant.

**Figure S11, related to Figure 2 and 6.** Related to Figure 2, **(A)** circulating IgG antibodies to both the vaccine and challenge virus with or without DT treatment at 4 days post infection**.** Data are from one experiment with 5 male mice per group. Related to Figure 6, **(B)** circulating IgG antibodies to both the vaccine and challenge virus with or without DT treatment at 4 days post infection**.** Data are from one experiment with 3-4 male mice per group. Related to Figure 2, **(C)** Circulating IgG antibodies to both the vaccine at 4 days post infection at a plasma dilution of 1:100 for females mice (n=8/10; combined from two independent experiments) and male mice (n= 23/25; combined from five independent experiments).

**Figure S12, related to Figure 1D.** Representative flow cytometry gating strategy for CD19+ B cells and CD3+ T cells. C57BL/6J lung cells were isolated as described in methods. Gating was performed by singlet (FSC-A v FSC-H), leukocytes (removing high SSC-A and low SSC-A/FSC-A), live cells. Values shown as frequency (%) of parent population.

**Figure S13, related to Figure 1D-E.** Representative flow cytometry gating strategy for NKp46+ NK1.1+ Natural Killer (NK) cells. C57BL/6J lung cells were isolated as described in methods. Gating was performed by singlet (FSC-A v FSC-H), leukocytes (removing high SSC-A and low SSC-A/FSC-A), live cells. Values shown as frequency (%) of parent population.

**Figure S14, related to Figure 2B. (A)** Representative flow cytometry gating strategy for depletion of NKp46+ NK1.1+ Natural Killer (NK) cells with diphtheria toxin (DT). NKp46-DTR lung cells were isolated as described in methods. Gating was performed by singlet (FSC-A v FSC-H), leukocytes (removing high SSC-A and low SSC-A/FSC-A), live cells. Values shown as frequency (%) of parent population.

**Figure S15, related to Figure 5A-C.** Representative flow cytometry gating strategy for CD3+ T cells and their activation via CD69, and CD3+CD4+ and CD3+CD8+ T cells.. NKp46-DTR lung cells were isolated as described in methods. Gating was performed by singlet (FSC-A v FSC-H), live cells, and leukocytes (removing high SSC-A and low SSC-A/FSC-A). Values shown as frequency (%) of parent population.

**Figure S16, related to Figure 5D-F.** Representative flow cytometry gating strategy for CD19+ B cells, Ly6C-high (hi) ‘inflammatory’ monocytes, and Ly6G+ Neutrophils. NKp46-DTR lung cells were isolated as described in methods. Gating was performed by singlet (FSC-A v FSC-H), live cells, and leukocytes (removing high SSC-A and low SSC-A/FSC-A). Values shown as frequency (%) of parent population.

**Supplementary Tables:**

Table S1: Methods of NK depletion during acute influenza infection.

| **Author** | **Mouse Strain** | **Mouse Sex** | **Flu Strain** | **Dose of Flu Challenge** | **NK Depletion** | **Main Findings** |
| --- | --- | --- | --- | --- | --- | --- |
| (Gazit et al., 2006) | 129/Sv, C57BL/6 | Not specified | A/PR/8/34 (H1N1) | 3x10^3^ PFU  (or 0.12 HAU),  60-70% survival in WT | NCR1-gfp knockout | Loss of NCR1 (NKp46) results in lethal infection (0% survival) |
| (Zhou et al., 2013) | C57BL/6 | Female | A/PR/8/34 (H1N1) | 5 HAU - 0% survival (in WT) 0.5 HAU - 30% survival (in WT)  0.0625 HAU - 80% survival (in WT) | Anti NK1.1* | High dose (5 HAU) infection + NK cell depletion improves survival.  Medium dose (0.5 HAU) infection + depletion decreases survival.  Low dose (0.0625 HAU) + depletion has no effect. |
| (Abdul-Careem et al., 2012) | C57BL/6 BALB/C | Male | A/PR/8/34 (H1N1) | 5x10^3^ PFU (i.n.)  0% survival (in WT) | IL-15-/- KO mice, anti-NK1.1*, anti-asialo GM1^†^ | NK cells depleted with anti-NK1.1 or anti-asialo GM1, and IL-15-/-KO mice had increased survival and lower weight loss compared to WT |
| (Nakamura et al., 2010) | C57BL/6 | Not specified | Mouse-adapted A/FM/1/47 (H1N1) | 1000 PFU (i.n.)  0% survival in WT | IL-15-/- KO mice^‡^ | IL-15 deficiency improved survival of virus infected mice |
| (Stein-Streilein and Guffee, 1986) | B6D2F1 | Female | A/PR/8/34 (H1N1) | 0.125 HAU (intratracheal) 50% survival in WT mice. | Anti-asialo GM1^†^ | NK cell depleted mice (and hamsters) had decreased survival to infection and increased viral titers |
| (Nogusa et al., 2008) | C57BL/6 | Male | A/PR/8/34 (H1N1) | 10^4^ TCID50 HAU | Anti-NK1.1* | Mice depleted of NK cells lost significantly more weight than infected WT young mice and had greater viral loads. |
| (Kos and Engleman, 1996) | C57BL/6 | Female | A/WSN | 1000 HAU (i.p) | Anti-NK.1.1* | NK cells were essential for the induction of influenza virus-specific CTL responses |

Note: Such methods of NK cell depletion have been shown not to be specific. *NK1.1 is also used as a marker of NKT cells and ^†^anti-asialo GM1 can not only recognize T cells, but administration can also lead to basophil depletion (Nishikado et al., 2011). ^‡^IL-15 KO mice, while primarily exhibiting an NK cell deficiency, also suffer from T cell deficiencies and impaired lymphocyte trafficking (Lodolce et al., 1998;Itsumi et al., 2009). Therefore, we used NKp46 expression as the basis for NK cell depletion, as it has been shown to be expressed by mouse NK cells (Biassoni et al., 1999), but also some type 1 innate lymphoid cells (Cortez and Colonna, 2016).

**Supplementary Figures:** Figure S1:


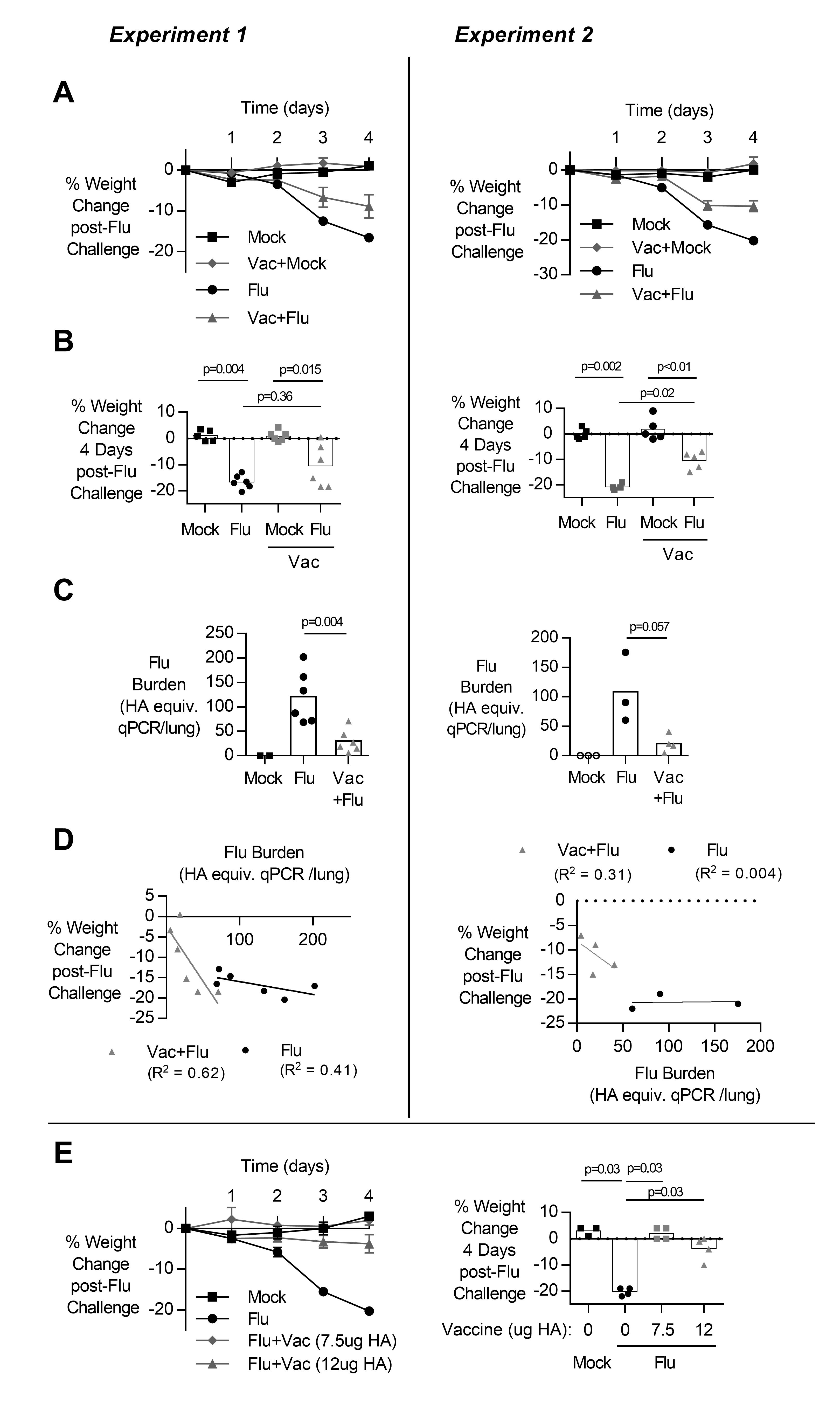


Figure S2:


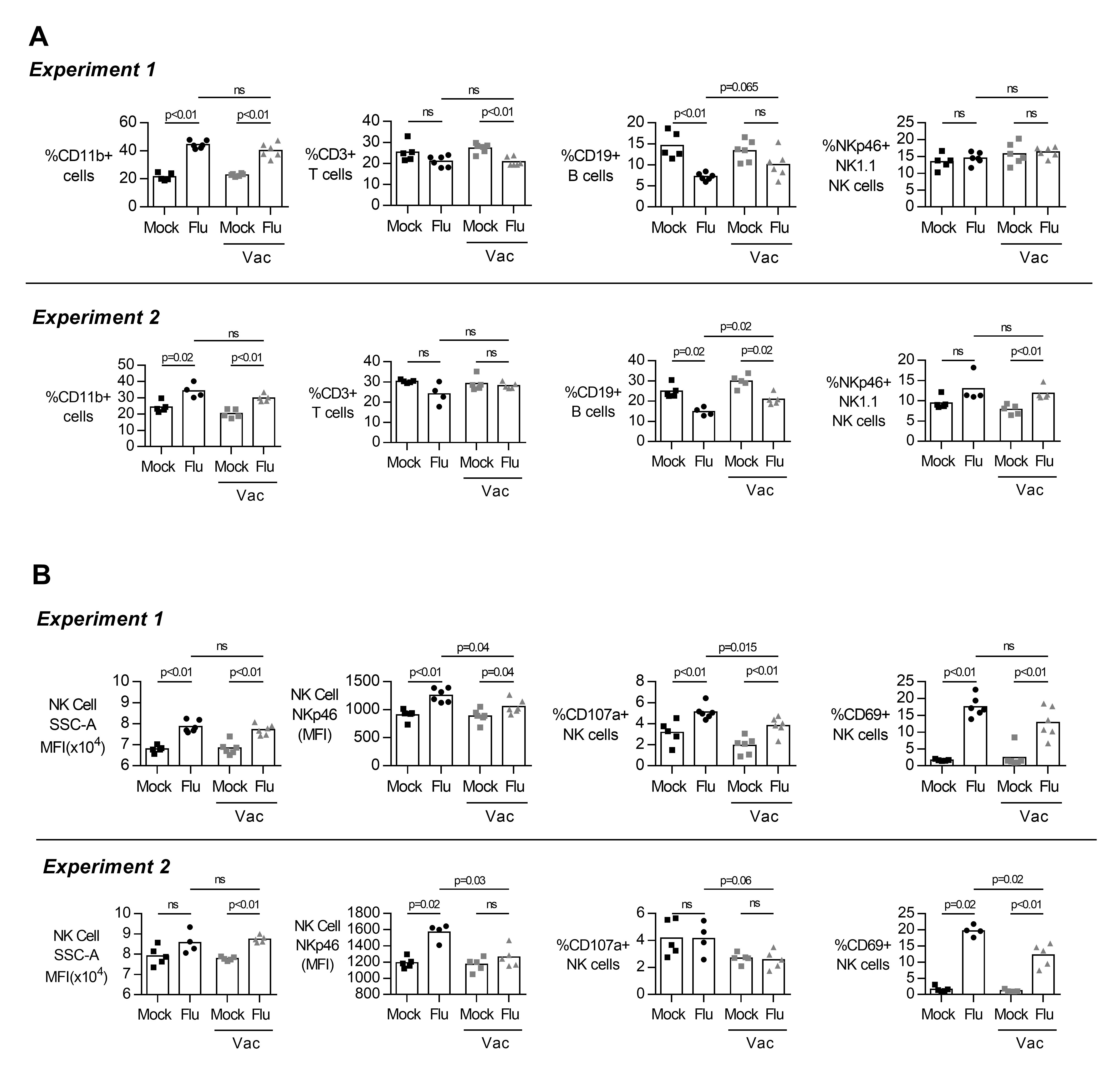


Figure S3:


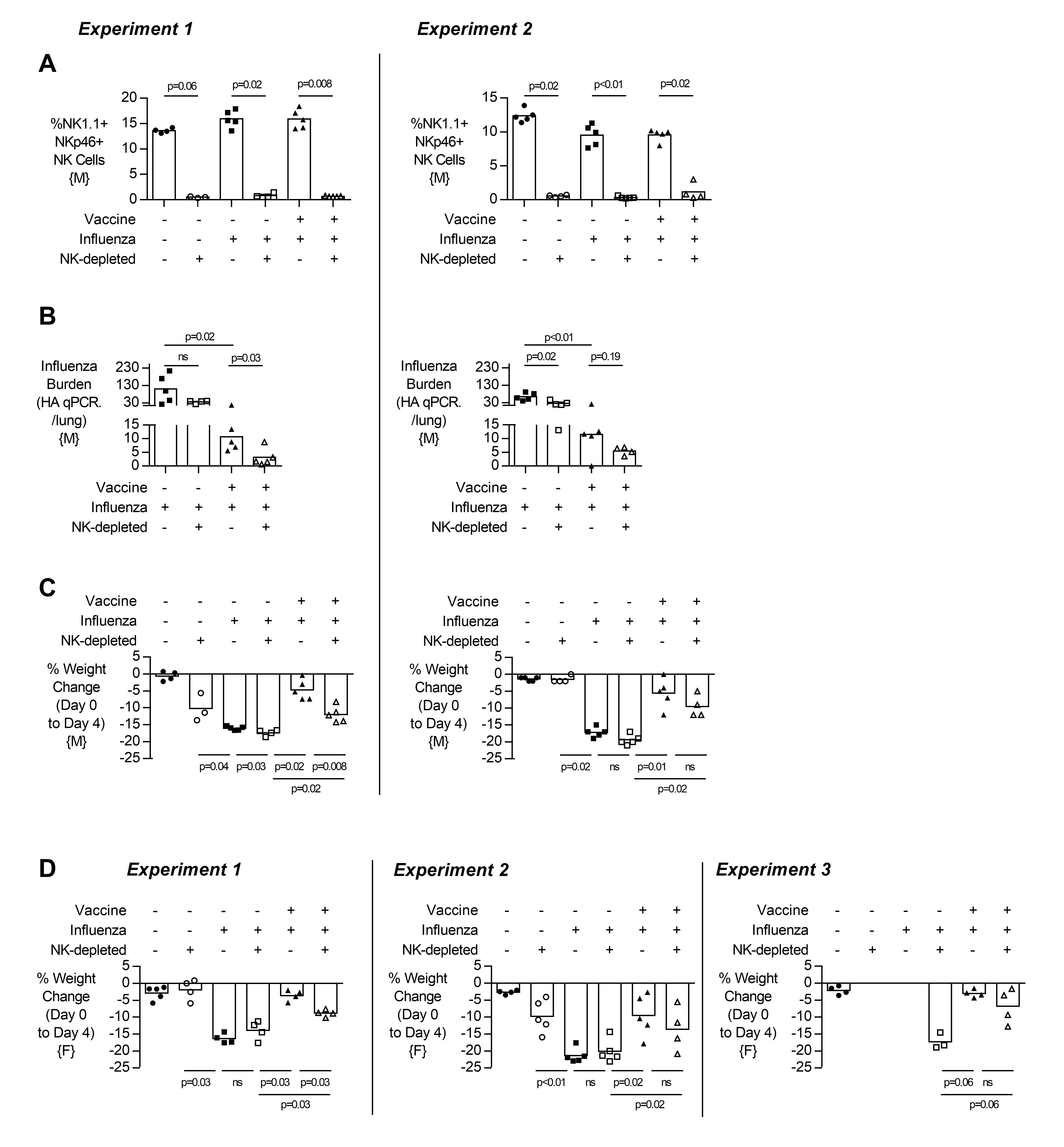


Figure S4:


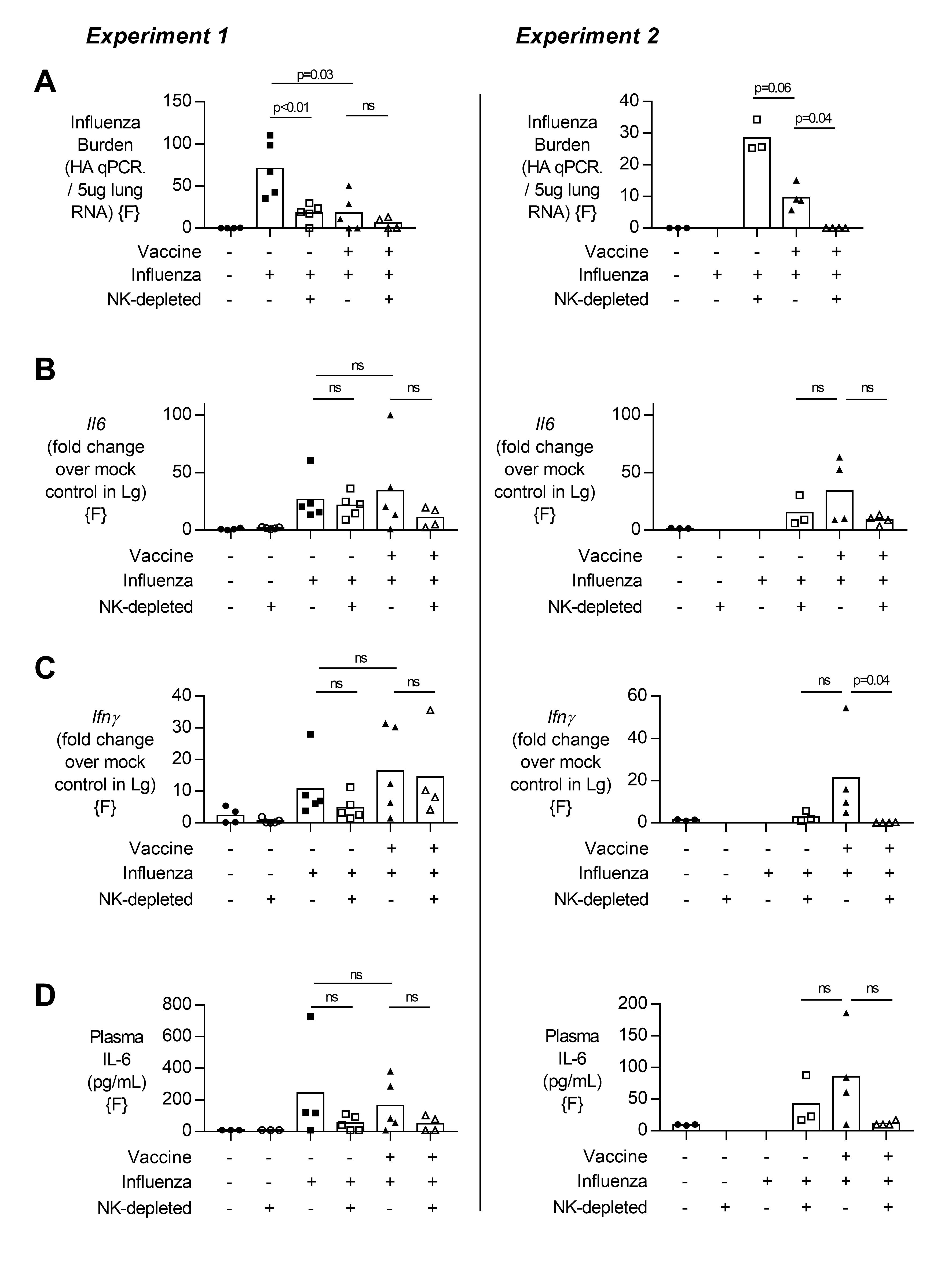


Figure S5:


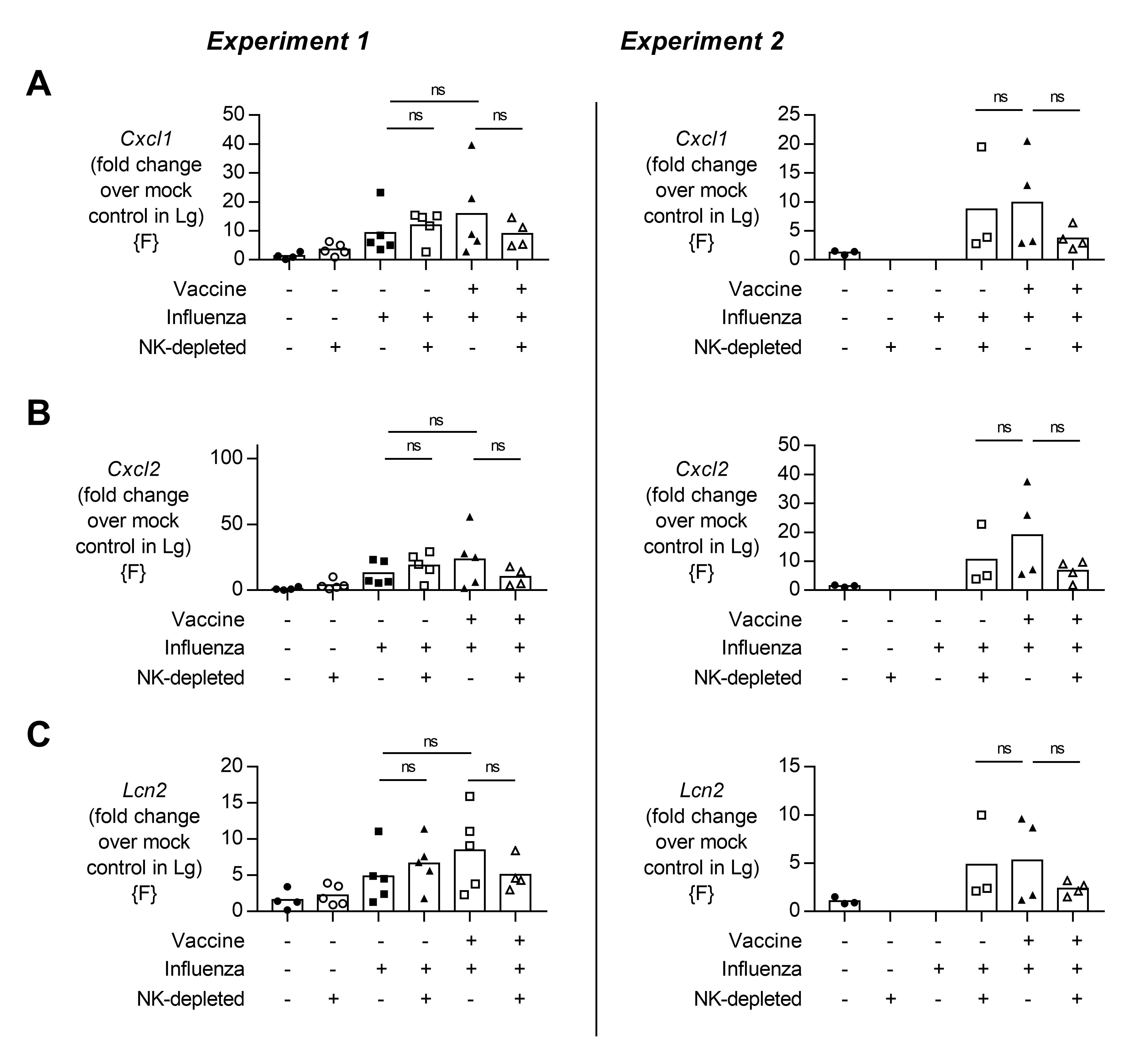


Figure S6:


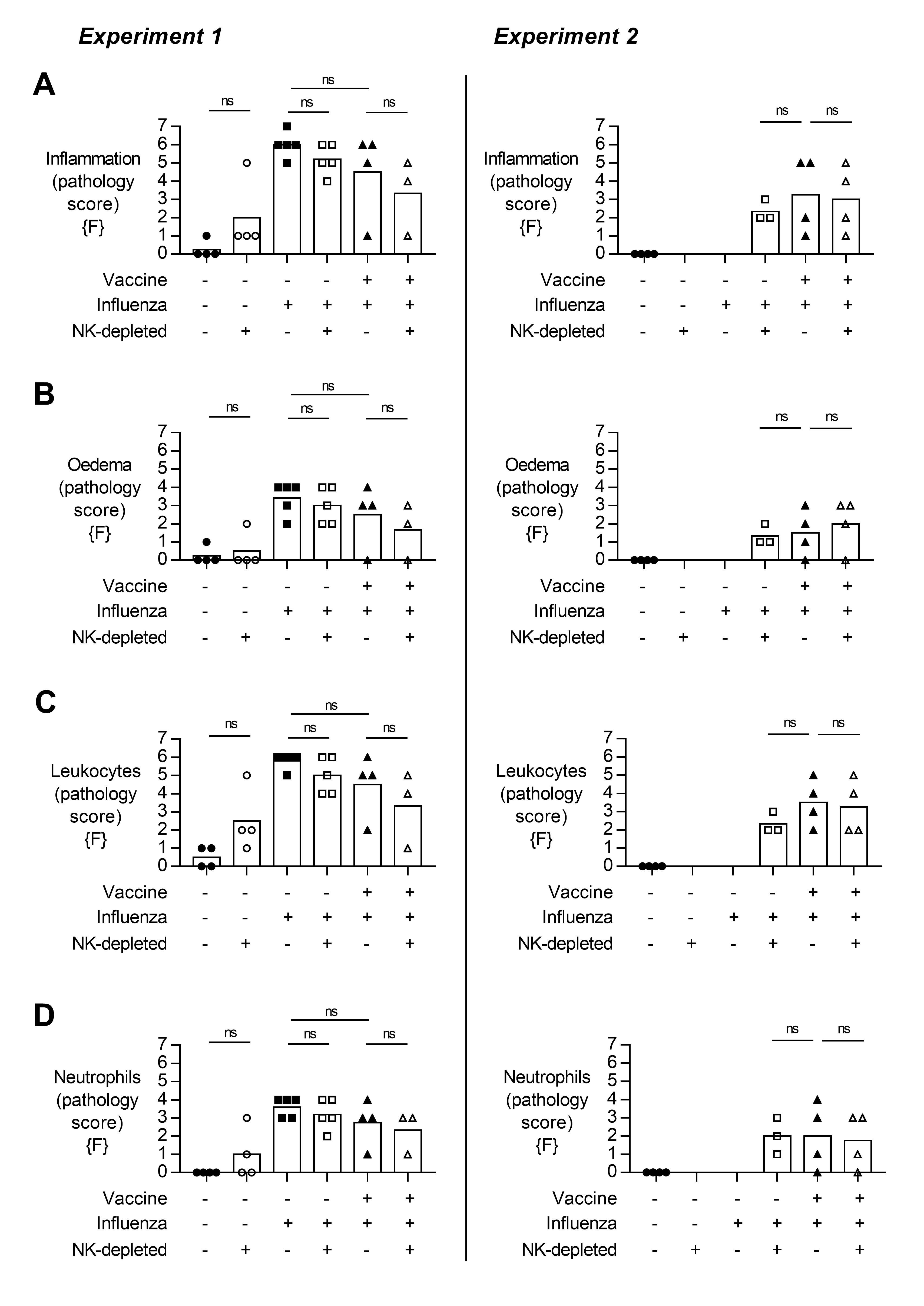


Figure S7:


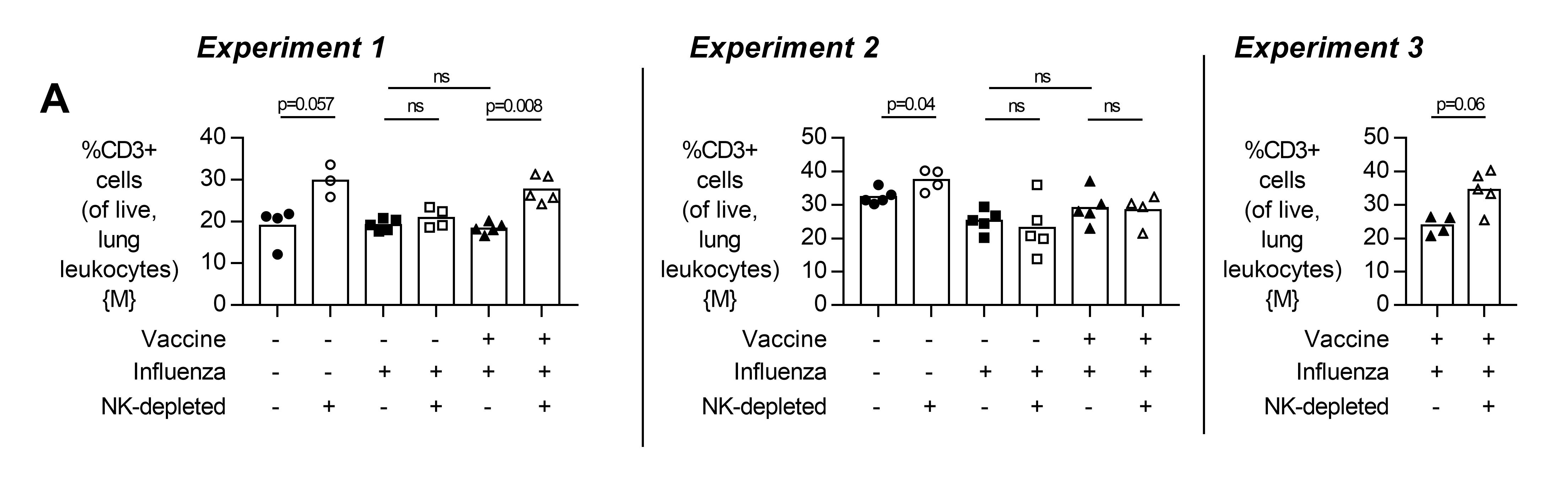


Figure S8:


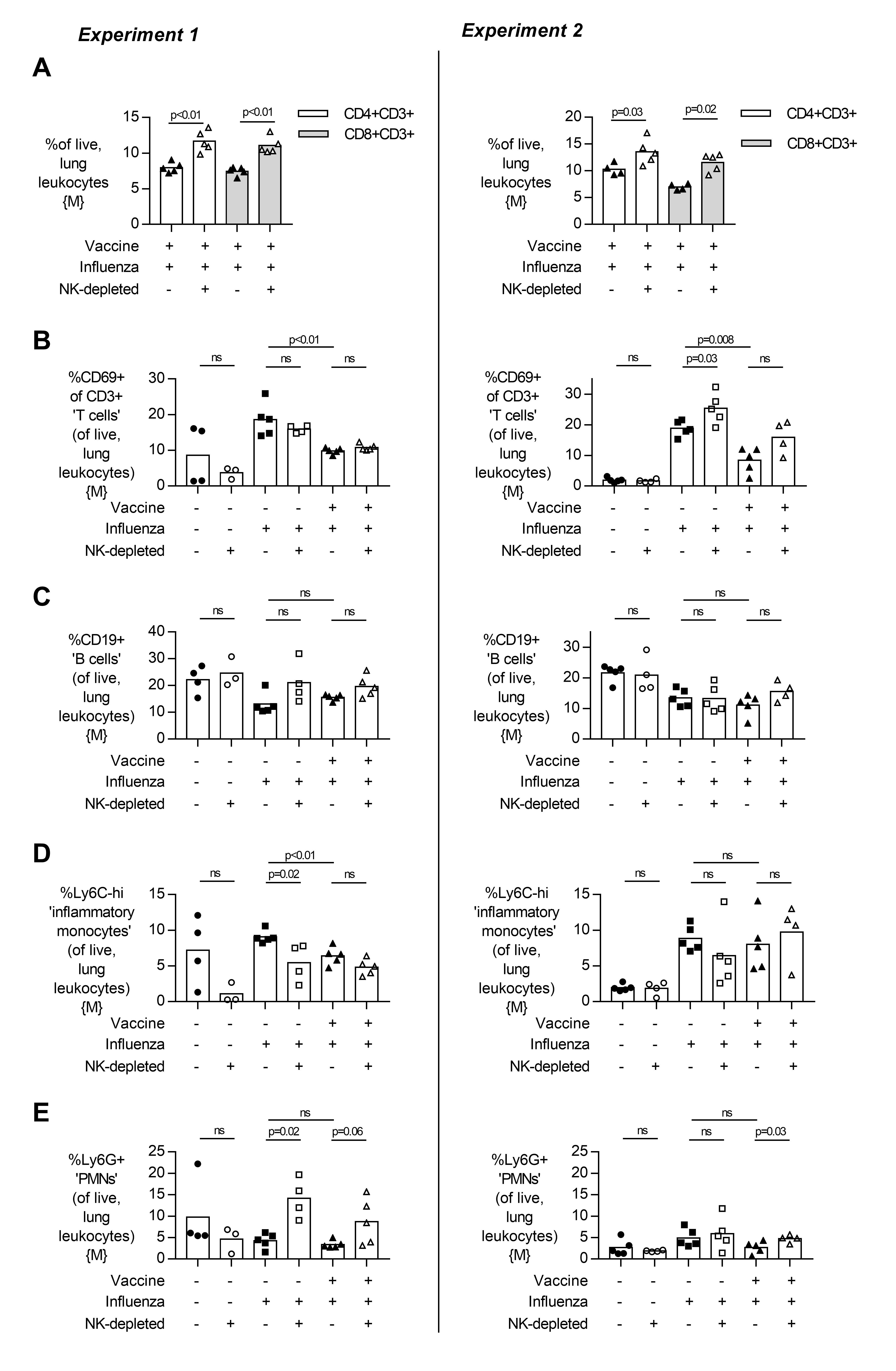


Figure S9:


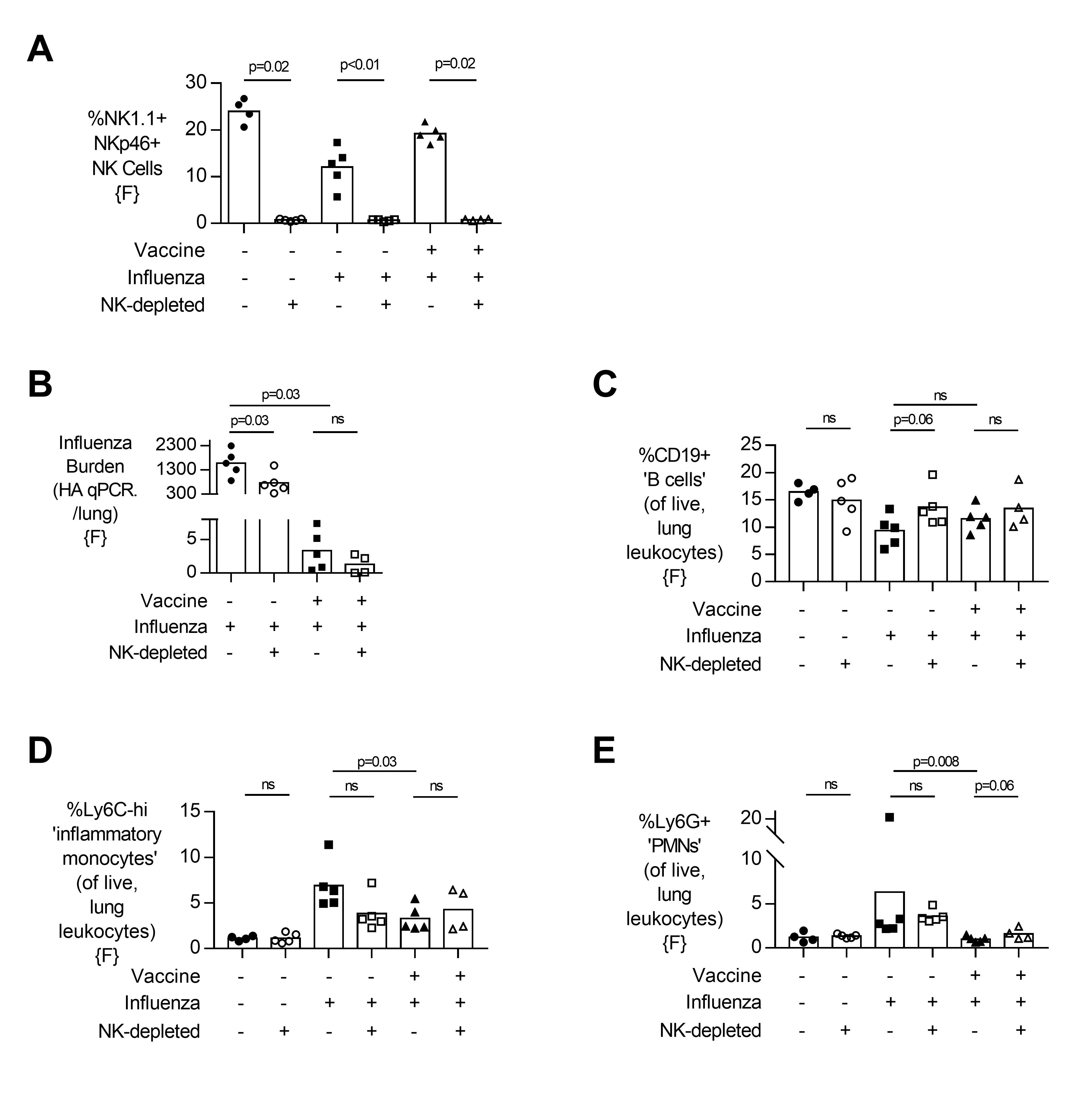


Figure S10:


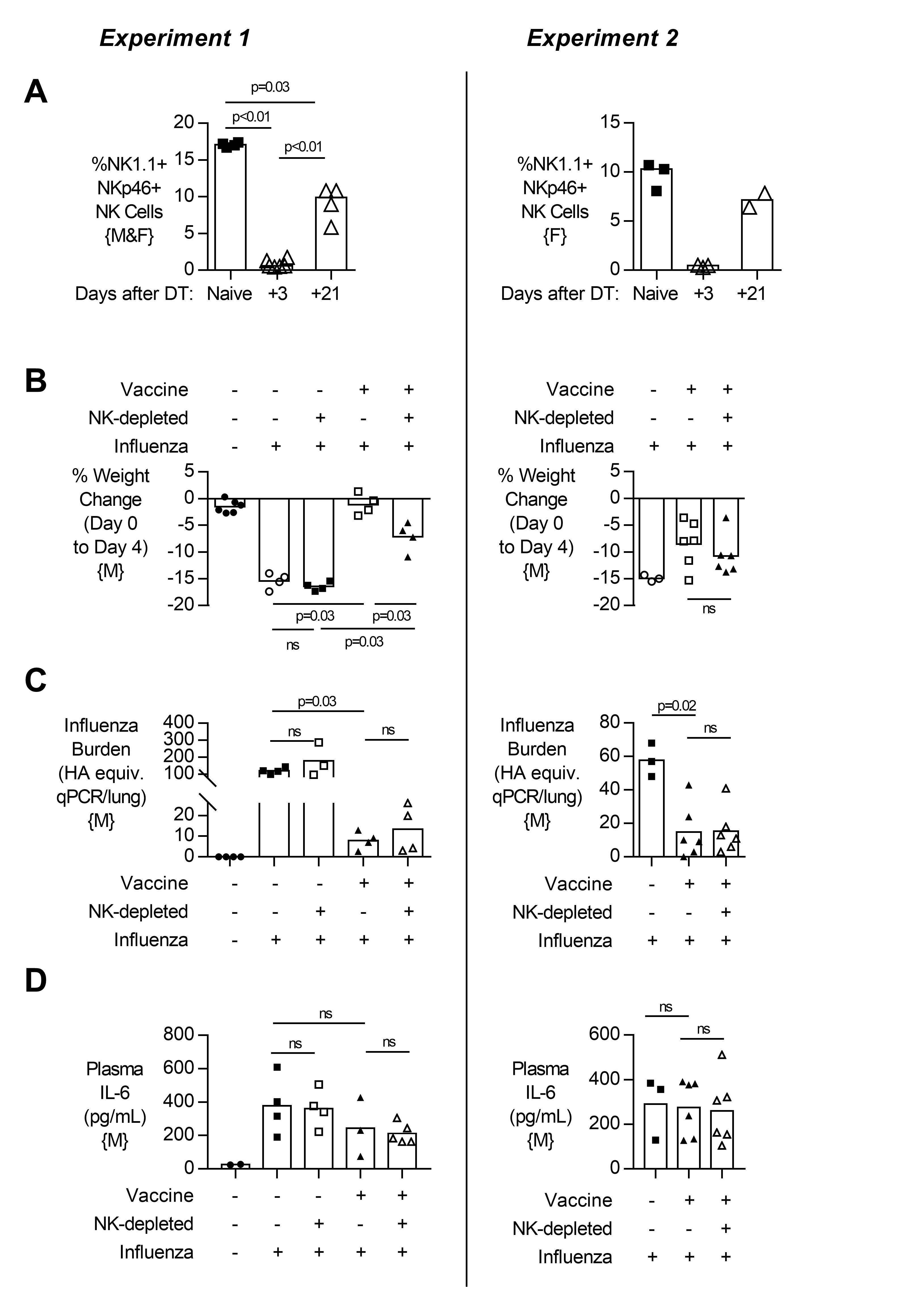


Figure S11:


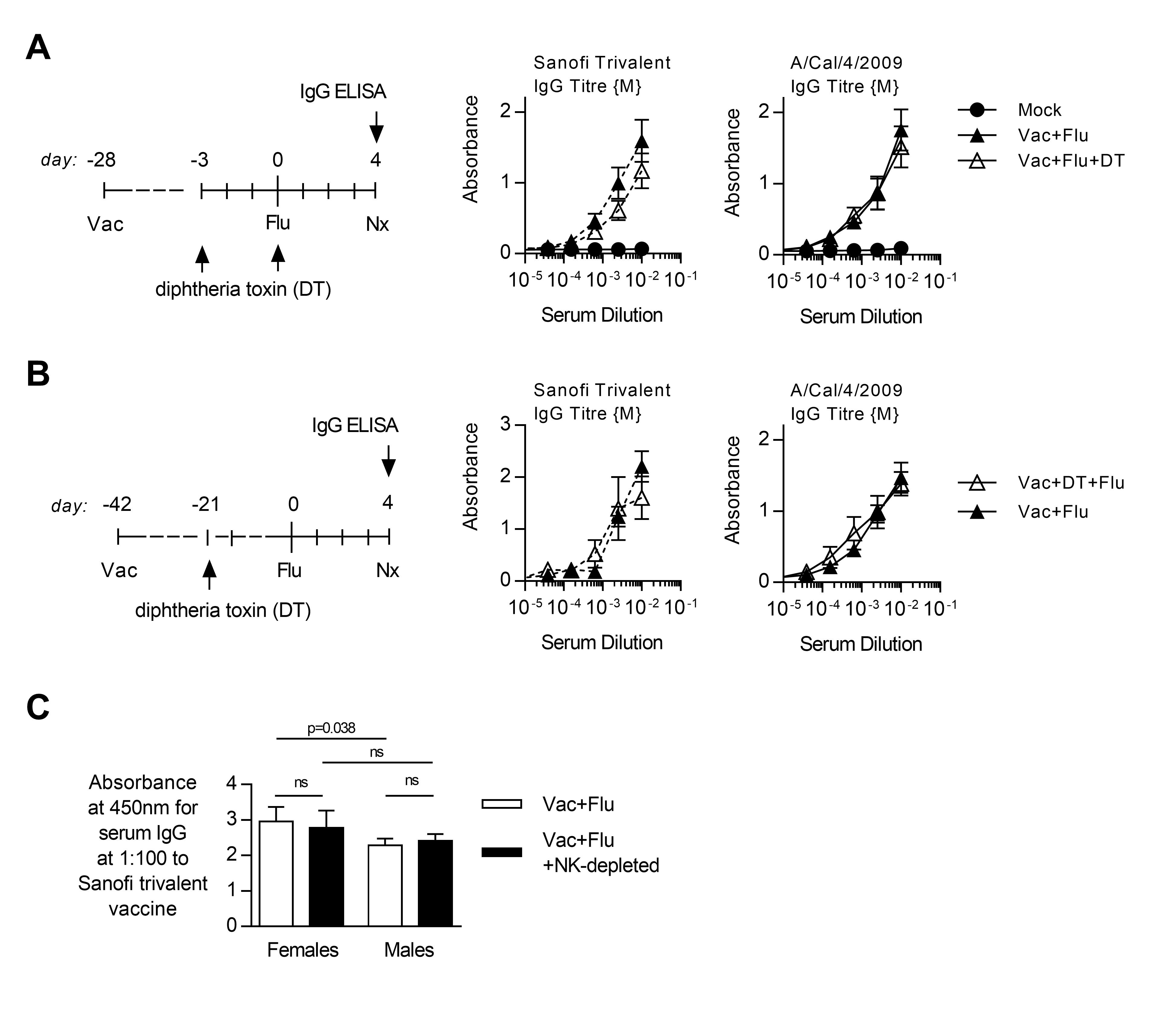


Figure S12:


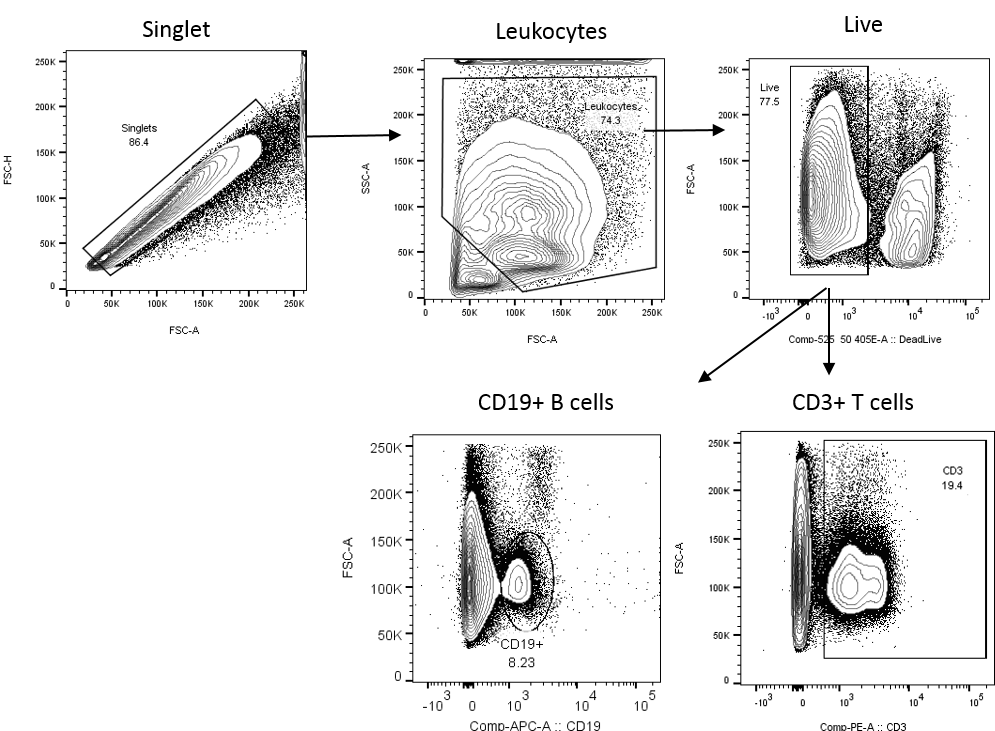


Figure S13:


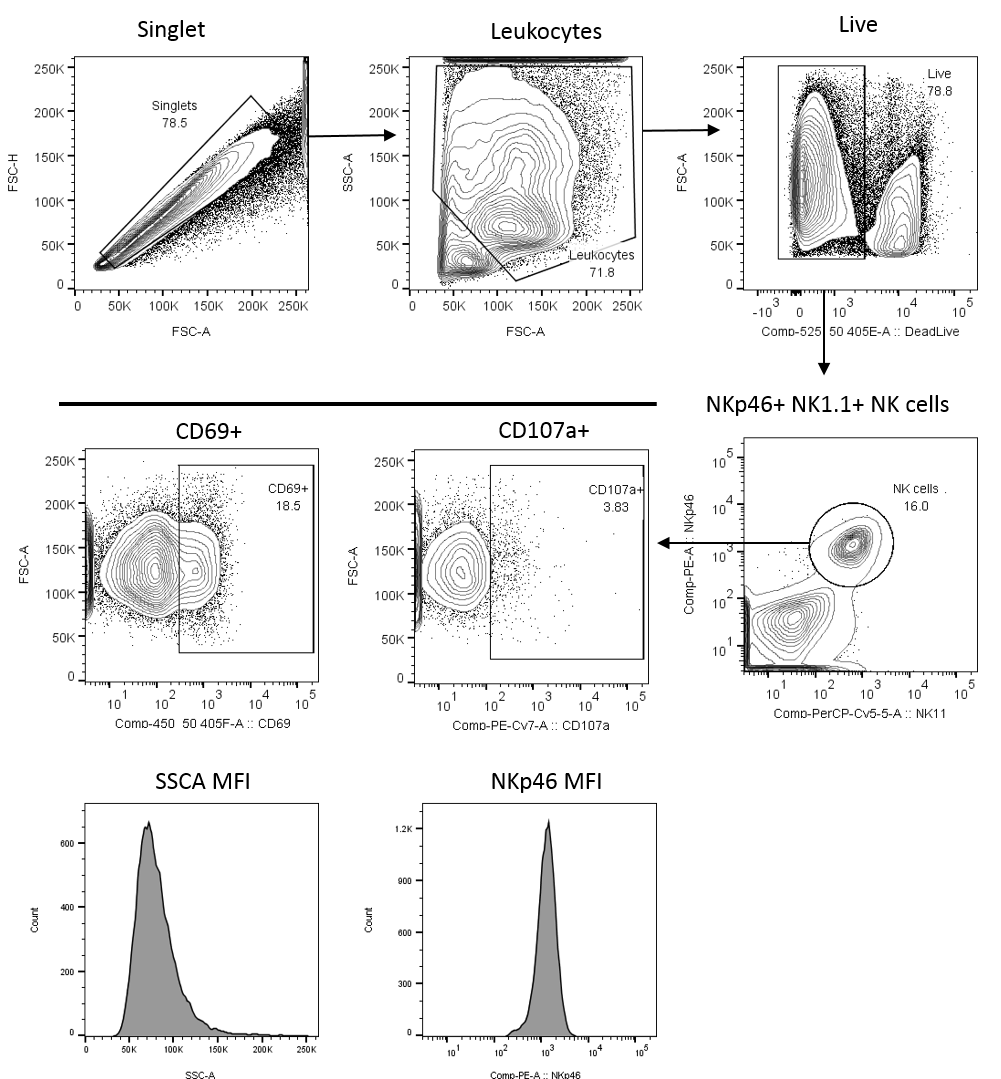


Figure S14:


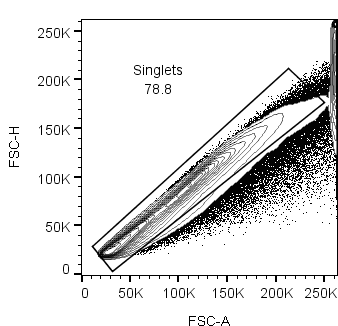

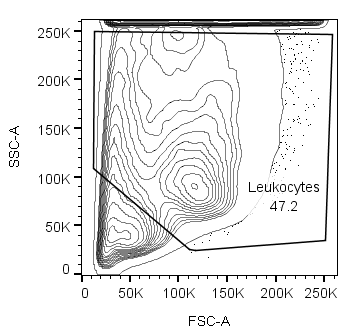

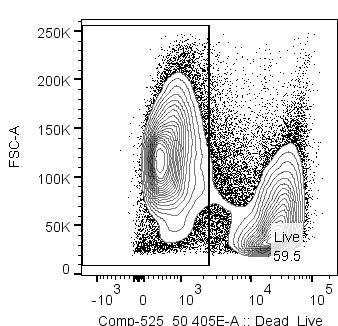

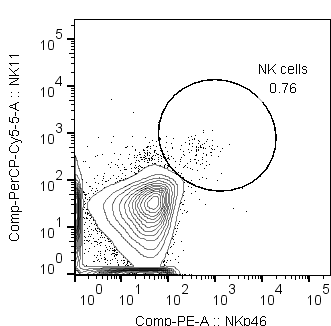

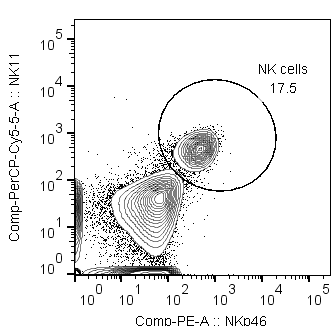


Vac+Flu

Vac+Flu+DT

Singlet

Live

Leukocytes

**A**

Figure S15:


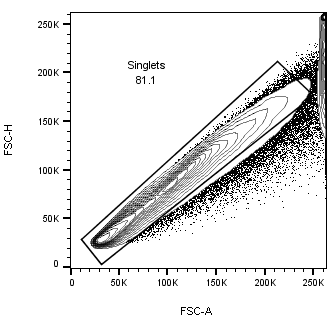

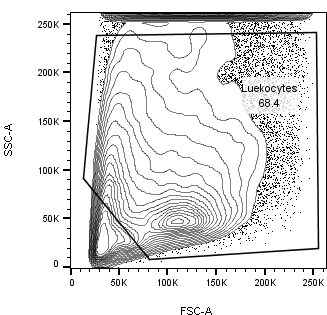

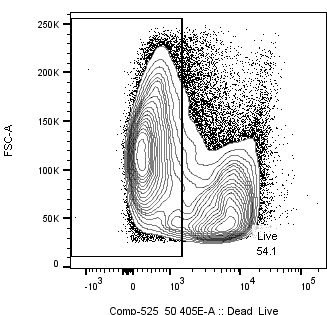

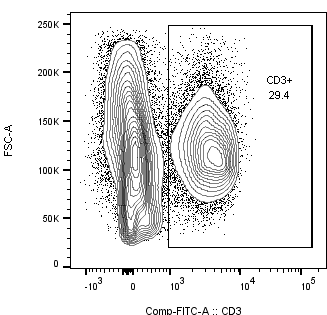

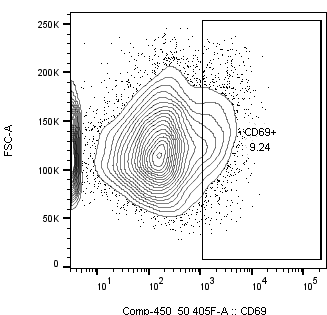

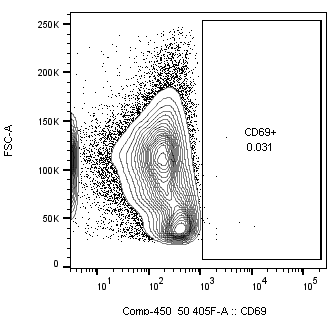

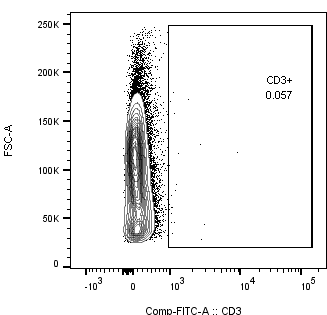


Singlet

Leukocytes

CD3 T cells

Leukocytes

CD69+

**Unstained Gates:**

CD3

CD69


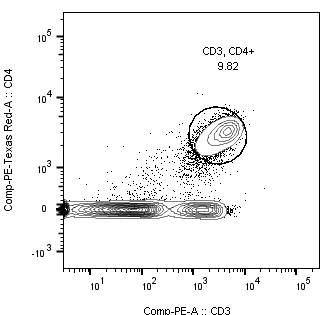

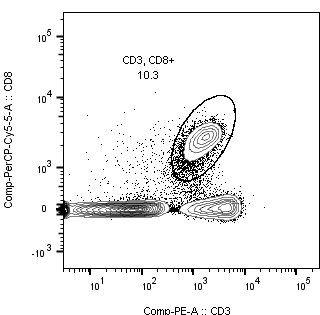


CD4+ CD3+

CD8+ CD3+

Figure S16:


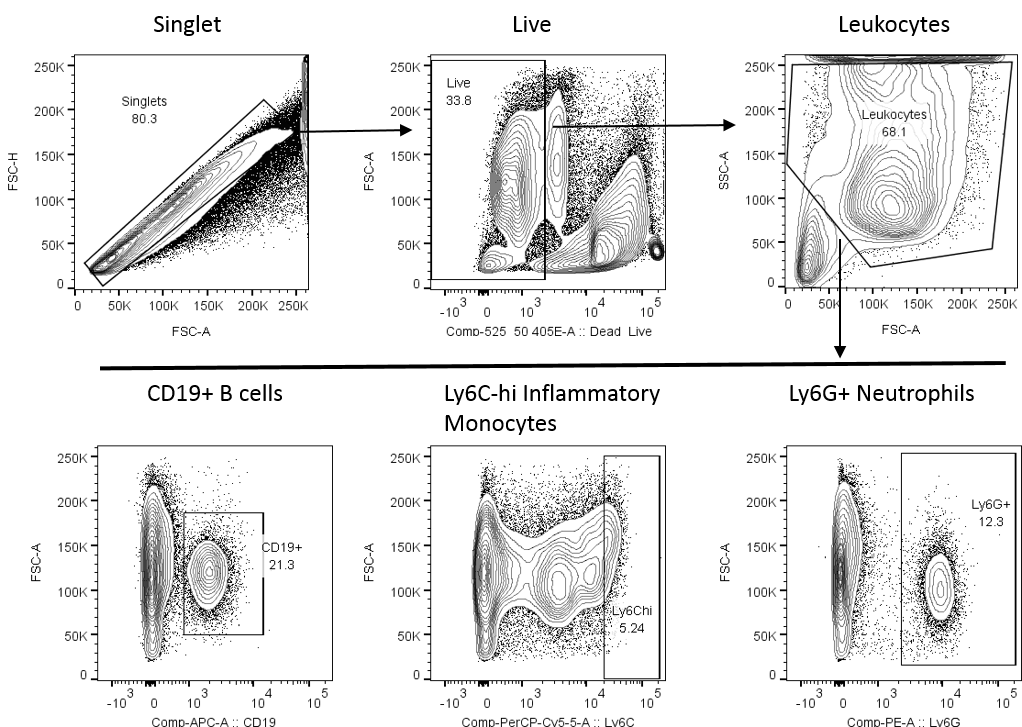

Supplement: Supplementary file 1 [file Data_Sheet_1.docx]
